# Editing of rice *PSEUDO-ETIOLATION IN LIGHT* microProtein genes promotes chloroplast development

**DOI:** 10.1093/plcell/koaf235

**Published:** 2025-10-07

**Authors:** Heebak Choi (최희백), Tae Gyu Yi (이태규), Yun-Shil Gho (고윤실), Ki-Hong Jung (정기홍), Sun-Hwa Ha (하선화)

**Affiliations:** Graduate School of Green-Bio Science, College of Life Sciences, Kyung Hee University, Yongin 17104, Republic of Korea; Graduate School of Green-Bio Science, College of Life Sciences, Kyung Hee University, Yongin 17104, Republic of Korea; Graduate School of Green-Bio Science, College of Life Sciences, Kyung Hee University, Yongin 17104, Republic of Korea; Graduate School of Green-Bio Science, College of Life Sciences, Kyung Hee University, Yongin 17104, Republic of Korea; Graduate School of Green-Bio Science, College of Life Sciences, Kyung Hee University, Yongin 17104, Republic of Korea

## Abstract

The rice (*Oryza sativa*) PSEUDO-ETIOLATION IN LIGHT (OsPEL) microProtein family members function as dominant-negative regulators of chloroplast development and are conserved among land plants. Knockout of all 3 *OsPEL* genes enhanced plant greening traits and was accompanied by leaf anatomical modifications associated with chloroplast-enriched bundle sheath cells in rice. These phenotypic changes correlated with increased CO_2_ assimilation efficiency and yield. OsPEL1 specifically interacts with key positive regulators of photosynthesis, the rice GOLDEN2-LIKE (OsGLK) transcription factors and the PHOTOSYSTEM I ASSEMBLY 2 (OsPSA2) chaperone. OsPEL1 inhibits these regulators by sequestering OsGLK1 and OsPSA2 in the cytoplasm, which prevents their proper localization to the nucleus and chloroplast, respectively. Supported by RNA-seq evidence of transcriptional homeostasis in greening-related genes, we reveal a multilayered regulatory mechanism and identify the OsPEL family as a promising target for crop improvement.

## Teaser

Loss of function of OsPEL microProteins promotes chloroplast development by relieving their cytoplasmic sequestration of OsGLK1 and OsPSA2, enhancing photosynthetic efficiency and yield in rice.

## Introduction

A key challenge in preserving stable crop productivity is the improvement of the photosynthesis process, which has been actively explored through regulation of chloroplast development, chlorophyll biosynthesis, and subcellular structures (Eva et al. 2019; Simkin et al. 2019; Baslam et al. 2020). In nature, C_4_ and Crassulacean acid metabolism represent advantageous photosynthetic models that exhibit high CO_2_ fixation rate under diverse CO_2_ levels, high temperature, and low humidity (Edwards 2019; Ferrari and Freschi 2019 ). Various strategies, such as reconstructing existing enzymatic pathways, optimizing photosynthesis, and creating new photosynthetic systems, have been applied to introduce C_4_ photosynthesis mechanism into C_3_ crop plants (Eva et al. 2019; Simkin et al. 2019; Ermakova et al. 2020; Croce et al. 2024). Rice (*Oryza sativa* L.), a major C_3_ crop plant that feeds 50% of the human population and provides 21% of energy and 15% of protein per capita, has been considered an ideal target for the implementation of C_4_ photosynthesis for over 50 years (Jeong et al. 2017; Ermakova et al. 2020).

A notable progress in making C_4_ rice was achieved by increasing the level of GOLDEN2-LIKE (GLK) transcription factor, a positive regulator for chloroplast development (Wang et al. 2013; Choi et al. 2024). Several studies to overexpress 2 maize *GLKs* (*ZmGLK1/ZmG1/golden2* and *ZmG2*) in rice resulted in the formation of proto-Kranz structure, an initial step toward C_4_ photosynthesis evolution, alongside increases in chlorophyll contents, enhanced chloroplast development, improved photosynthetic efficiency, and higher grain yield (Wang et al. 2017; Ermakova et al. 2020; Li et al. 2020; Yeh et al. 2022). However, in rice, attempts to enhance chloroplast development through the FOX (full-length cDNA overexpressor) mutation of *OsGLK1*, corresponding to its overexpression, were restricted to nongreen cells, with no effects observed in green cells where the majority of photosynthesis takes place (Nakamura et al. 2009). It can be inferred that a robust posttranslational counteracting mechanism exists for rice GLKs, serving as a primary obstacle to enhancing chloroplast development. On another note, the development of chloroplasts is highly dependent on photosystem (PS) I and II as key components of the photosynthetic apparatus. A chloroplast-localized chaperone called *PHOTOSYSTEM I ASSEMBLY 2* (*PSA2*), which is a DnaJ-type zinc finger protein, is required for the structural stability of PSI (Fristedt et al. 2014). In maize and *Arabidopsis*, loss-of-function mutation in *PSA2* resulted in a pale-green phenotype, suggesting a crucial role for PSA2-mediated thiol transaction in forming a complex with PsaG in the thylakoid lumen (Fristedt et al. 2014; Wang et al. 2016).

In the pursuit of targets for enhancing photosynthesis through genome editing, the *Arabidopsis PSEUDO-ETIOLATION IN LIGHT* (*AtPEL1*) gene (*AT3G55240*), identified to reduce chlorophyll content when activated (Ichikawa et al. 2006), has emerged as highly promising. Furthermore, a carrot ortholog of *PEL* (*DCAR_032551*) was characterized as a carotenoid accumulation trait gene (*Y*) that negatively influences carotenoid deposition in taproots (Iorizzo et al. 2016 ). In rice, the *DEEP GREEN PANICLE1* (*DGP1/OsPEL1*), a panicle-specific negative regulator of chlorophyll synthesis, was shown to enhance chlorophyll content in glumes when knocked out while leaving the chlorophyll levels in leaves unaffected (Zhang et al. 2021). Recent studies on *Arabidopsis PELs* have further revealed significant insights into their regulatory roles: 3 *REPRESSOR OF PHOTOSYNTHETIC GENEs* (*RPGEs/PELs*) negatively affect photosynthesis (Kim et al. 2023a ); 4 PELs negatively regulate both chlorophyll and anthocyanin accumulation (Han et al. 2023); and *BRZ-INSENSITIVE-PALE GREEN 4* (*BPG4/RPGE2/PEL1*) is implicated in chloroplast homeostasis via light and brassinosteroid (BR) signaling (Tachibana et al. 2024). Across these studies, knockout mutants consistently exhibited higher chlorophyll levels than the wild type, but additional phenotypes were also observed: increased seed yields in the *rpge1/rpge2/rpge3* triple mutant (Kim et al. 2023a), a minor reduction in biomass in the *pel1/pel2/pel3/pel4* quadruple mutant (Han et al. 2023), and elevated reactive oxygen species (ROS) generation in the *bpg4* single mutant leading to photosynthetic damage under high-light conditions (Tachibana et al. 2024). Meanwhile, the suppressing target of a carrot RPGE/PEL has been identified as an *ARABIDOPSIS PSEUDO-RESPONSE REGULATOR2-like* (*APRR2*-like) transcription factor (*DcAPRR2*), which mediates transcriptional activation of carotenogenic genes (Wang et al. 2024). In common, these finding indicate that the molecular mechanism of PEL/RPGE/BPG proteins predominantly involves inhibition of the DNA-binding properties of transcription factors, primarily GLKs, 6 MYB (e.g. AtMYB4), and APRR2. This suggests the broad roles of PEL proteins as negative regulators for greening traits and pigment accumulation, including chlorophylls, anthocyanins, and carotenoids (Han et al. 2023; Wang et al. 2024; Yokoyama 2024).

In this study, we employed genome editing to target the PEL family, identified as broad negative regulators of greening-associated traits, with the goal of improving agricultural productivity through enhanced photosynthesis. By confirming the direct physical interactions of OsPEL1 with candidate proteins, including OsGLKs (transcription factors) and OsPSA2 (a molecular chaperone), predicting 3D interaction models, and proposing common molecular mechanisms for inhibiting their functions in the cytoplasm, we elucidated the molecular basis of enhanced greening traits in triple PEL knockout rice plants with leaf anatomical features indicating increased chloroplast accumulation near vascular tissues. These findings suggest that rice can tolerate stressful conditions due to enhanced photosynthesis resulting from the absence of PEL genes. Furthermore, this study advances our understanding of the homeostatic mechanisms inherent in plants that regulate greening traits.

## Results

### Diversification of PEL microProteins reflects the evolutionary history of land plants

**Figure 1.**
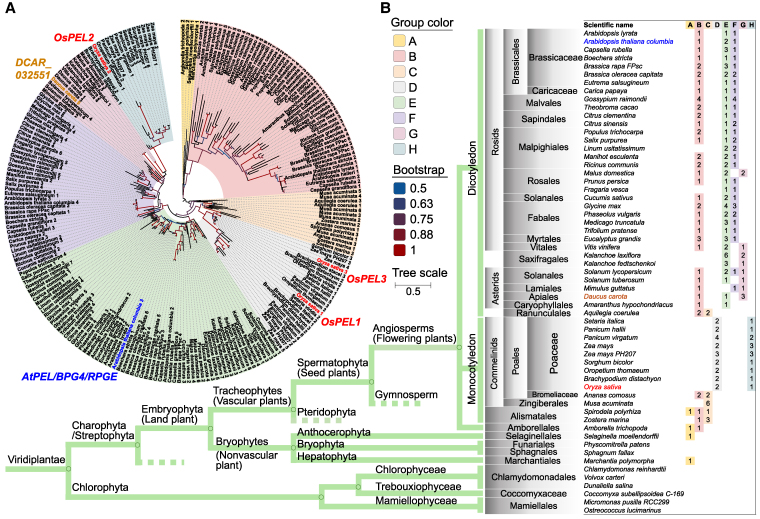
The phylogenetic tree of PEL family among 64 species revealing evolutionary history. **A)** The schematic view of phylogenetic tree of 227 genes from 64 species classified into 8 groups. **B)** The 8 groups of the PEL family were rearranged on the basis of their evolutionary order. The thick green line represents the cladogram of the evolution including green plants (Viridiplantae). The species that showed similar group patterns within the same genus (*Arabidopsis lyrata*, *Capsella grandiflora*, *Setaria viridis*, *Brachypodium stacei*, and *Micromonas pusilla CCMP1545* showed the same pattern with *Arabidopsis halleri*, *Capsella rubella*, *Setaria italica*, *Brachypodium distachyon*, and *Micromonas pusilla RCC299*, respectively) were not shown.

Three rice PELs, namely, OsPEL1 (LOC_Os01g62060, 102 a.a.), OsPEL2 (LOC_Os03g17200, 78 a.a.), and OsPEL3 (LOC_Os05g38680, 102 a.a.), have been identified as microProteins due to their small size and negative regulatory function (Eguen et al. 2015; Bhati et al. 2021). From 64 photosynthetic species (phytozome v.12.1.6), 227 PELs were detected with an evolutionary relevance by phylogenetic tree (Fig. 1; Supplementary Data Sets S1 to S3). In single-celled photosynthetic species, none of the PEL homologs exist. The most ancient group is A, which was found in Bryophytes (nonvascular plant). The B group first branched out, probably due to whole-genome duplication, when the Tracheophytes (vascular plants) evolved. It resides throughout the Angiosperms (flowering plants), including monocot plants, except for Poaceae and most dicot plants. Next, it diverged into C group, consisting of monocot-preferential group, except Poaceae. The coexistence of both B and C group PEL homologs in *Aquilegia coerulea*, a representative of the basal eudicot lineage (Kramer 2009), suggests that the divergence of these gene groups occurred early in angiosperm evolution, likely prior to the radiation of core eudicots. The newly emerged D and H groups comprise Poaceae-specific groups, and the E, F, and G groups have been intensively diversified in dicot plants in terms of multiple copies and combinations, forming dicot-specific groups. On the phylogenetic tree, OsPEL1 and OsPEL3 belong to the same D group and OsPEL2 belongs to H group, suggesting a well conservation within Poaceae species of monocot plants. Meanwhile, *Arabidopsis* and carrot species have 4 and 5 PELs including AtPEL/BPG4/RPGE2 (Ichikawa et al. 2006) and DCAR_032551 (Iorizzo et al. 2016) in common with E and independently F or G basically with B group. It is proposed that the transition of the PEL family might have a significant impact on the evolution process of terrestrial plants.

### OsPEL1, OsPEL2, and OsPEL3 redundantly act as negative regulators of greening traits

**Figure 2.**
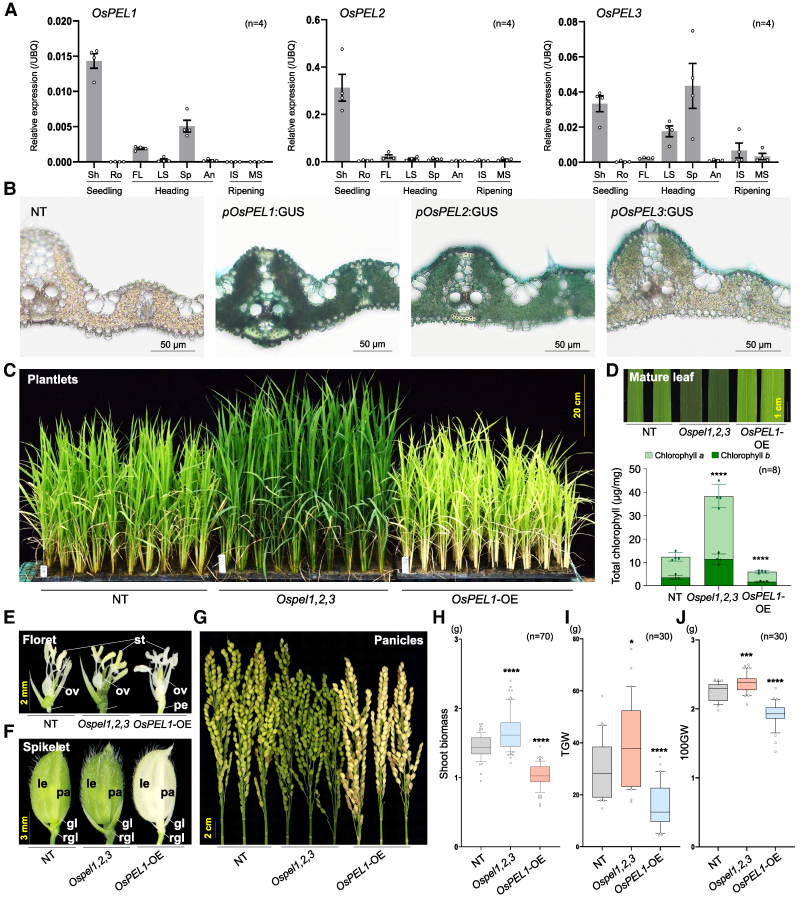
Spatiotemporal expression of the *OsPEL* family and phenotypic and agronomic analysis. **A)** Relative expression levels of *OsPEL1*, *OsPEL2*, and *OsPEL3* in various tissues and development stages determined by RT-qPCR. (Sh, shoot; Ro, root; FL, flag leaf; LS, leaf sheath; Sp, spikelet; An, anther; IS, immature seed; MS, mature seed; *n* = 4; the bars represent the standard error of the mean.). **B)** GUS reporter assay using 2 kb promoter regions from 3 *OsPEL* genes. **C)** Whole plant phenotypes from complete disruption of *OsPEL* family (*Ospel1,2,3*) and *OsPEL1* overexpression (*OsPEL1*-OE) compared to NT plant. **D)** Green color phenotypes in the middle region of mature leaf blades and their chlorophyll contents (*n* = 4; the bars represent the standard error of the mean, ****P < 0.0001, Student's *t-*test). **E** to **G)** Greening phenotype of floral organs. Florets including stamen (st), ovary (ov), and pedicel (pe) **(E)**; spikelets comprising palea (pa), lemma (le), glumes (gl), and rudimentary glume (rgl) **(F)**; and overview of panicles **(G)**. **H** to **J)** Agronomic traits from field-grown T3 generation plants, including shoot biomass (*n* = 70) **(H)**, TGW (*n* = 30) **(I)**, and 100GW (*n* = 30) **(J)**. Other 6 agronomic traits that showed no significant changes in *Ospel1,2,3* line are presented in Supplementary Fig. S5. Statistical significance is indicated as *P < 0.05, ***P < 0.001, and ****P < 0.0001 based on Student's *t*-test. For elements of the boxplots, center line, median; box limits, upper and lower quartiles; whiskers, 10% to 90%; and points, outliers.

To understand the physiological functions of OsPELs in rice plants, we investigated their spatial and cell-type-specific expression patterns, along with their subcellular localization. First, transcript levels of the 3 *OsPEL* genes were examined across various tissues (Fig. 2A). All 3 genes showed dominant expression in young shoots, suggesting a shared role in green tissue function. To analyze cell-type specificity in leaves, GUS reporter assays using the 2 kb upstream promoter regions of each *OsPEL* gene were performed in stable transgenic rice plants (Fig. 2B). These assays revealed that the *OsPEL1* promoter drives the strongest GUS activity, predominantly in mesophyll (MS) cells—parenchyma cells that serve as the main site of photosynthesis—and in the epidermal layers, which are typically devoid of chloroplasts. The *OsPEL2* promoter exhibited relatively high expression in MS cells and the adaxial epidermis, whereas *OsPEL3* promoter activity was restricted to the adaxial epidermis. Although weak, both *OsPEL1* and *OsPEL2* promoters showed detectable expression in bundle sheath (BS) cells. Subcellular localization of each OsPEL-GFP fusion protein was assessed via transient expression, revealing that all 3 OsPEL proteins localize to both the nucleus and cytoplasm (Supplementary Fig. S1A), implying potential functional redundancy among the family members. To confirm this localization pattern, OsPEL1 was stably overexpressed, revealing GFP signals in both the nucleus and cytoplasm—most strongly in vascular bundles, which are largely chloroplast deficient—followed by BS and MS cells, along with reduced chlorophyll autofluorescence, indicating biological activity of the OsPEL1-GFP fusion protein (Supplementary Fig. S1B). Collectively, these findings indicate that the 3 OsPELs are broadly expressed across leaf tissues, not only in photosynthetically active cells but also in those lacking chloroplasts, suggesting their overlapping and fine-tuned roles in regulating greening traits in rice, supported by their shared subcellular localization.

To reverse-genetically investigate the physiological functions of OsPELs, we constitutively overexpressed each of the 3 *OsPEL* genes with a C-terminal Myc tag (OsPEL-OEs). All 3 overexpression lines exhibited similar phenotypes, including pale-green coloration across most tissues and a significant reduction in chlorophyll content compared to nontransgenic (NT) rice plants—49.3% in *OsPEL1*-OE, 39.5% in *OsPEL2*-OE, and 41.5% in *OsPEL3*-OE—suggesting overlapping functions in regulating chlorophyll accumulation (Supplementary Fig. S2). For the loss-of-function studies, we initially generated 24 to 84 single CRISPR lines for *OsPEL1*, *OsPEL2*, and *OsPEL3* (Supplementary Fig. S3). Based on visual observation, these single knockout lines displayed no consistent or significant alterations in leaf color, suggesting probable functional redundancy. To test for functional redundancy among the 3 genes, we employed a multiplexed CRISPR/Cas9 system (Xie et al. 2015) to simultaneously target all 3 *OsPEL* genes. This strategy produced a range of edited T0 plants (*n* = 71), including single, double, and triple knockouts (Supplementary Fig. S4, A to C). Following self-pollination, we obtained 4 stable triple knockout (*Ospel1,2,3*) lines in the T1 generation. These lines exhibited markedly dark-green leaves and a 48% to 79% increase in chlorophyll content compared to NT plants (Supplementary Fig. S4, D and E). Backcrossing of one line (C49#1) confirmed the redundancy of *OsPEL* gene*s*, as the dark-green phenotype and elevated chlorophyll levels consistently cosegregated with the triple KO genotype (Supplementary Fig. S4F). In the T3 generation, the *Ospel1,2,3* and *OsPEL1*-OE lines displayed contrasting phenotypes, with dark-green and pale-green leaf coloration, respectively (Fig. 2C). In flag leaves, these color changes corresponded to a 3.1-fold increase and a 0.5-fold decrease in chlorophyll content for the *Ospel1,2,3* and *OsPEL1*-OE lines, respectively, relative to NT plants (Fig. 2D). Similar phenotypic trends were observed in reproductive organs, including florets (stamens, ovaries, and pedicels; Fig. 2E), spikelets (palea, lemma, glumes, and rudimentary glumes; Fig. 2F), and panicles (bundles of spikelets and uppermost internodes; Fig. 2G).

These greening phenotypes were directly reflected in biomass, with the *Ospel1,2,3* mutants showing a 1.2-fold increase and the *OsPEL1*-OE lines a 0.7-fold decrease compared to NT plants (Fig. 2, C and H). In terms of agronomic performance, *Ospel1,2,3* lines exhibited enhanced yield traits, with a 30.9% increase in total grain weight (TGW) (Fig. 2I) and a 5.3% increase in 100-grain weight (100GW) (Fig. 2J), without significant negative effects on other agronomic traits (Supplementary Fig. S5). Conversely, *OsPEL1*-OE lines showed substantial reductions across multiple traits: 47.0% in TGW, 14.6% in 100GW, 19.8% in filling rate, 6.4% in panicle length, and 22.5% in culm length under normal growth conditions (Fig. 2, I and J; Supplementary Fig. S5). These results reveal an inverse correlation between *OsPEL* gene expression and photosynthetic capacity and yield.

### Loss of function in the *OsPEL* family promotes chloroplast development, resulting in enhanced photosynthesis in rice

**Figure 3.**
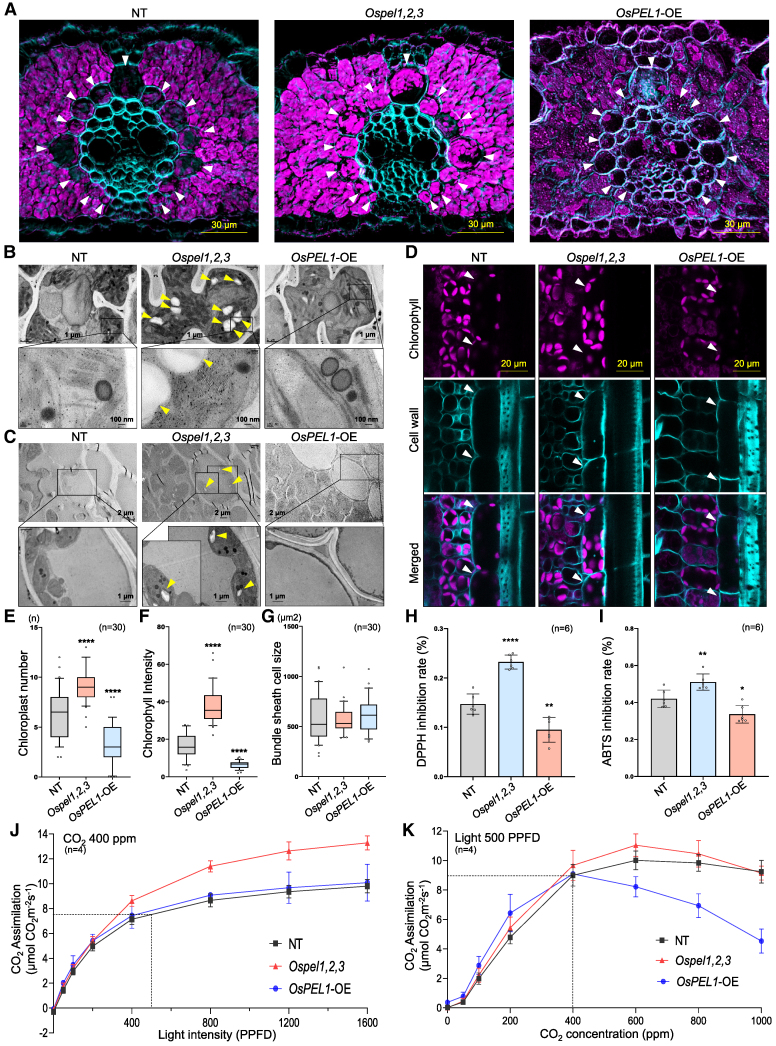
Increased chloroplast development in the *Ospel1,2,3* and its impact in enhanced CO_2_ assimilation under varying light and CO_2_ concentration conditions. **A)** Super-resolution microscopy images of the transverse section of leaf blades from NT, *Ospel1,2,3*, and *OsPEL1*-OE lines, presented as merged views. BS cells are indicated by white arrows. In the *Ospel1,2,3* line, chloroplasts were specifically enriched in BS cells, exhibiting anatomical characteristics distinct from those typically observed in C_3_ plants. Single-channel images are provided in Supplementary Fig. S6, and 3D rotation movies are available in Video 1 to 3. **B** and **C)** TEM images of MS cells **(B)** and BS cells **(C)** from NT, *Ospel1,2,3*, and *OsPEL1*-OE lines. Starch granules, observed exclusively in *Ospel1,2,3*, are marked with yellow arrowheads. **D)** Confocal microscopy images of vertically dissected leaves for the autofluorescence signal of chlorophylls from NT, *Ospel1,2,3*, and *OsPEL1*-OE lines. BS cells are marked with white arrows. **E** to **G)** Quantification of BS cell parameters shown in **(D)**: chloroplast number **(E)**, chlorophyll intensity **(F)**, and cell size per each cell **(G)**. **H** and **I)** ROS scavenging activities measured by DPPH **(H)** and ABTS **(I)** inhibition assays. Statistical significance in **(E** to **I)** is indicated as *P < 0.05, ***P < 0.001, and ****P < 0.0001 based on Student's *t*-test. And the bars represent the standard error of the mean. **J** and **K)** Photosynthetic rates were measured as net CO_2_ assimilation (μmol CO_2_ m^−2^s^−1^) under fixed CO_2_ concentration with varying light intensities **(J)** and fixed light intensity with varying CO_2_ concentrations **(K)** in field-grown plants at 80 d after germination. For elements of the boxplots, center line, median; box limits, upper and lower quartiles; whiskers, 10% to 90%; and points, outliers.

To investigate the anatomical changes at the cellular levels, we performed 3 types of microscopies. Transverse section images obtained via super-resolution microscopy (SRM) revealed markedly enhanced chlorophyll fluorescence in *Ospel1,2,3* compared to NT and *OsPEL1*-OE, indicating significantly promoted chloroplast development (Fig. 3A; Supplementary Fig. S6; Videos 1 to 3). Interestingly, chlorophyll fluorescence extended prominently into BS cells, in addition to MS cells, in *Ospel1,2,3* lines, whereas it remained largely confined to MS cells in NT plants. In contrast, *OsPEL1*-OE exhibited markedly weaker and collapsed fluorescence across all cell types, denoting the structurally destructed chloroplasts. These contrasting SRM results were well correlated with their transmission electron microscopy (TEM) imaging patterns, which showed highly and poorly developed grana stacks with an increased and decreased number of thylakoid membrane layers in both MS cells (Fig. 3B) and BS cells (Fig. 3C) between *Ospel1,2,3* and *OsPEL1*-OE, respectively. Also, increased starch granules in both MS and BS cells of *Ospel1,2,3* suggested higher levels of carbon assimilation than NT and *OsPEL1*-OE (Fig. 3, B and C). Further paradermal confocal images clearly showed the relatively abundant chloroplasts in BS cells of *Ospel1,2,3* and less in *OsPEL1*-OE leaves compared to few chloroplasts in NT (Fig. 3D). Statistical analyses for the number of chloroplasts (Fig. 3E) and chlorophyll intensity (Fig. 3F) showed 3.6- and 2.6-fold increases in *Ospel1,2,3* and 0.4- and 0.4-fold decreases in *OsPEL1*-OE compared to NT, respectively. Meanwhile, there were no significant differences in the cell size (Fig. 3G). Comprehensively, 3 microscopic observations for *Ospel1,2,3* consistently exhibited the enhanced chloroplast development in BS cells, which has been considered as a primary anatomical step for C_4_ photosynthesis evolution from C_3_ plant, as well as MS cells (Sage et al. 2014; Sedelnikova et al. 2018).

Considering a previous report that the *bpg4/rpge2/atpel* single mutation elevated ROS levels, leading to photosynthetic damage under high-light conditions in *Arabidopsis* (Tachibana et al. 2024), we measured ROS scavenging activity using 2,2-diphenyl-1-picrylhydrazyl (DPPH) and 2,2′-azino-bis (3-ethylbenzothiazoline-6-sulfonic acid) (ABTS) inhibition assays (Fig. 3, H and I). This analysis revealed a 1.6-fold and 1.2-fold increase in *Ospel1,2,3* lines and a 0.6-fold and 0.8-fold decrease in *OsPEL1*-OE lines, respectively, suggesting that enhanced chloroplast development via functional inactivation of the *OsPEL* family could lead to increased ROS scavenging potential in rice, unlike in *Arabidopsis*.

To assess the effect of enhanced chloroplast development on photosynthetic efficiency, we measured photosynthetic capacity under a fixed CO₂ concentration of 400 ppm, equivalent to normal atmospheric conditions (Fig. 3J). In *Ospel1,2,3* plants, photosynthetic capacity increased significantly from 21% at 400 photosynthetic photon flux density (PPFD) to 36% at 1,600 PPFD, confirming a direct contribution of developed chloroplasts to enhanced photosynthesis. The photosynthetic rate of *OsPEL1*-OE plants was much lower than that of *Ospel1,2,3* plants, but similar to NT plants across all light conditions. Given that chlorophyll content in *OsPEL1*-OE is approximately half that of NT plants (Fig. 2D), this suggests a potential feedback regulatory mechanism that compensates for impaired chloroplasts to maintain photosynthesis. Furthermore, under a fixed light intensity at 500 PPFD, photosynthetic rates were not significantly different among lines at low CO_2_ concentration. However, as CO₂ levels increased beyond 400 ppm, the differences became more pronounced—*Ospel1,2,3* plants showed up to a 10% increase at 600 ppm, while *OsPEL1*-OE plants exhibited a marked decrease, reaching 51% of the NT level at 1000 ppm (Fig. 3K). Consequently, the photosynthetic rate of *Ospel1,2,3* began to increase significantly at around 400 PPFD, whereas in *OsPEL1*-OE plants, it sharply declined beyond 400 ppm CO_2_ compared to the other 2 lines. These results indicate that *Ospel1,2,3* plants exhibit greater photosynthetic efficiency under high-light conditions than under elevated CO_2_, possibly due to enhanced chloroplast development that enables better utilization of excess light while leaving room for further improvement in CO_2_ assimilation.

### OsPEL1 is directly bound by OsGLKs and OsPSA2, both enclosing it in a grasp-like structure

**Figure 4.**
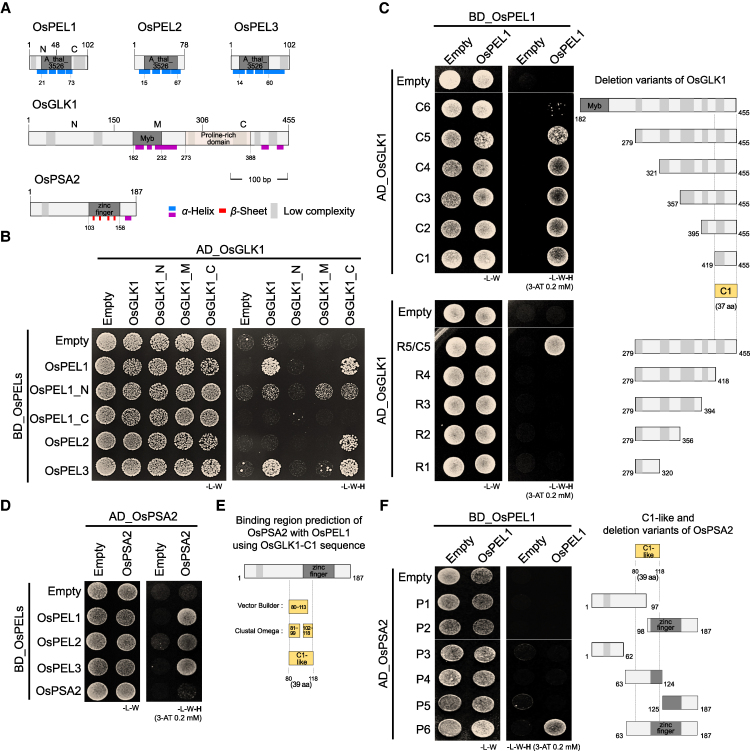
The Y2H analysis revealed the core binding regions of OsGLK1 and OsPSA2 for OsPEL1 interaction. **A)** Protein structures of the OsPEL family and its 2 binding targets, OsGLK1 and OsPSA2, are illustrated. **B)** Y2H experiments showing interactions between the OsPEL family and OsGLK family with fragmented proteins. **C)** Reciprocal Y2H experiments of OsPEL1 with detailed fragmentation of the OsGLK1 C-terminal region, focusing on the low complexity region. The cross-validated core binding motif, C1, is highlighted. **D)** Y2H experiments demonstrating interaction between the OsPEL family and OsPSA2. **E)** The OsPEL1-binding region within OsPSA2 was predicted by aligning the C1 region of OsGLK1 with the OsPSA2 using VectorBuilder and Clustal Omega alignment programs. **F)** Y2H experiments validating interactions between OsPEL1 and OsPSA2 fragments.

To elucidate the molecular mechanism of rice PELs, we identified 13 candidates interacting proteins through yeast 2-hybrid (Y2H) screening and validated 5 reliable protein partners (Supplementary Fig. S7). Among these, we focused on 2 kinds of proteins known to function as positive factors of greening traits, including chloroplast development and photosynthesis: One is OsGLK family, plant-specific GARP transcription factors, and the other is OsPSA2, a pivotal PS I assembly-associated chaperone with a zinc finger domain (Fig. 4A).

First, the OsPEL-binding region of OsGLK1 was investigated using Y2H analysis with full or partial fragments between the OsPEL family and OsGLK1 (Fig. 4B). The results showed strong binding affinity in the C-terminal region of OsGLK1 (OsGLK1_C) with each full length of 3 OsPELs, indicating the significance of the N-terminal (OsPEL1_N) than the C-terminal half of OsPEL1. Also, the full length of OsGLK1 binds to OsPEL1 and OsPEL3 but not OsPEL2, suggesting a closer structural similarity between OsPEL1 and OsPEL3. To further narrow down the C-terminal interaction region of OsGLK1 with OsPEL1, 10 bidirectional deletion variants were created (C1 to C6 and R1 to R4) (Fig. 4C). The 37 amino acids at the very end of C-terminus of OsGLK1 (OsGLK1_C1) were identified as the core area required for interaction with OsPEL1. Furthermore, the Myb domain of OsGLK1 may potentially interfere with the interaction with OsPEL1 when structurally exposed.

Next, the interaction of OsPSA2 with rice PEL family was elucidated by Y2H using full-length regions (Fig. 4D). The results indicated that OsPSA2 binds redundantly to all 3 OsPELs without forming homodimers. The region responsible for OsPEL binding was expected by analyzing with C1 amino acid sequence of OsGLK1 using 2 programs, the VectorBuilder and Clustal Omega. The aligned sequences were presented in similar regions spanning from 80th to 118th amino acid position, corresponding to the 39 amino acids (OsPSA2_C1-like) (Fig. 4E). To narrow down the interaction region, OsPSA2 was fragmented into 6 parts, with 2 evenly cut fragments (P1 and P2), 3 pieces (P3 to P5), and 1 longer piece (P6) that includes the C1-like domain to the C-terminal area with a zinc finger domain (Fig. 4F). The interaction of only the last P6 fragment with OsPEL1 suggests that the entire region from the C1-like domain to the end of C-terminus is necessary for the binding.

**Figure 5.**
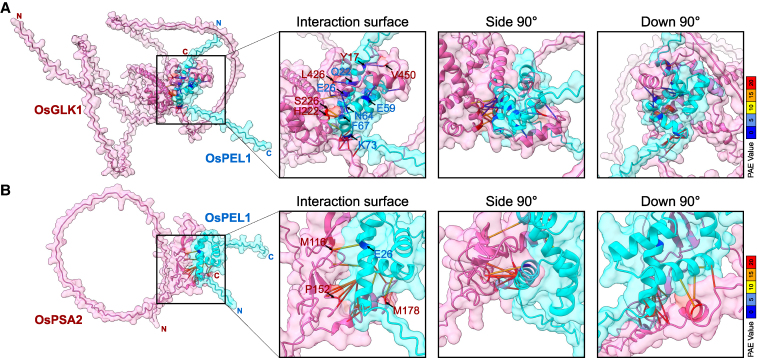
3D protein interaction modeling of OsPEL1 with both OsGLK1 and OsPSA2. **A)** 3D modeling of OsPEL1 and OsGLK1. **B)** 3D modeling of OsPEL1 and OsPSA2. Spinning movies of the 3D protein structures are available in Videos 4 and 5. The predicted core binding amino acids are marked in blue for OsPEL1 and in red for the target proteins. PAE values between the interacting amino acids are represented by pseudobonds, with colors ranging from blue (low) to red (high). Detailed information about the amino acids is provided in Supplementary Figs. S10 and S11.

From 3D modeling of OsPEL family (Supplementary Fig. S8), OsGLK1 and OsPSA2 (Supplementary Fig. S9), which provide basal protein structures, we have constructed 2 protein–protein interaction (PPI) complexes: OsPEL1–OsGLK1 (Fig. 5A; Supplementary Fig. S10; Video 4) and OsPEL1–OsPSA2 (Fig. 5B; Supplementary Fig. S11; Video 5). By using intersection data from “predicted aligned error (PAE)” values and “alanine scanning” analysis, the results reflect the precise prediction of the amino acid residues involved in PPI for both OsPEL1–OsGLK1 (Supplementary Fig. S10) and OsPEL1–OsPSA2 (Supplementary Fig. S11). As results, the predicted complex models proposed a tweezer-like structure formed by 2 α-helixes in the C-terminal region of OsGLK1 (Fig. 5A) and a hand-like structure formed by the zinc finger and an α-helix of OsPSA2 (Fig. 5B), which provide their structures to tightly hold the small ball-like OsPEL1. Furthermore, the PAE plot of OsGLK1 exhibited a distinct alteration following its interaction with OsPEL1, suggesting a significant increase in proximity between the Myb domain and the C-terminal region of OsGLK1 (Fig. 5A; Supplementary Fig. S9D). This finding implies that the DNA-binding activity of the Myb domain could be disrupted due to its interaction with OsPEL1, potentially affecting its regulatory functions.

### OsPEL1 interaction sequesters OsGLK1 and OsPSA2 in the cytoplasm, disrupting their subcellular localization

**Figure 6.**
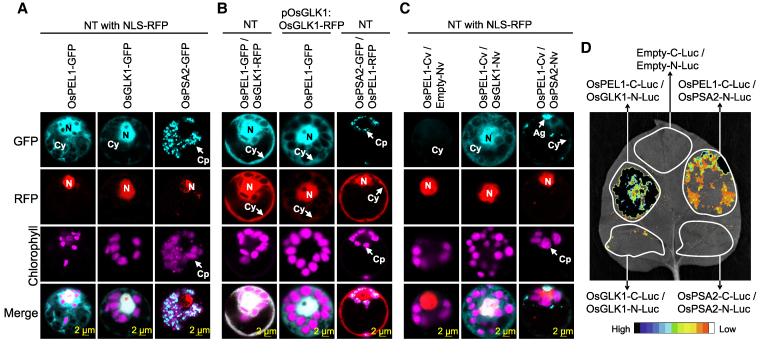
In planta localization and binding of OsPEL1 with OsGLK1 and OsPSA2. **A)** Subcellular localization of GFP-fused OsPEL1, OsGLK1, and OsPSA2 proteins in NT rice protoplasts, respectively. The nucleus localization marker (NLS-RFP) was cotransfected. **B)** Colocalization of OsPEL1 (as either OsPEL1-GFP or OsPEL1-RFP) with OsGLK1-RFP or OsPSA2-GFP in NT rice protoplasts. OsPEL1-GFP was also transfected into protoplasts isolated from pOsGLK1:OsGLK1-RFP transgenic plant. **C)** BiFC analysis between OsPEL1-Cv and 2 binding targets, either OsGLK1-Nv or OsPSA2-Nv. The nucleus localization marker (NLS-RFP) was cotransfected. The abbreviations and arrows indicate the nucleus (N), cytoplasm (Cy), chloroplast (Cp), and aggregates (Ag) in **A** to **C)**. **D)** Split-luciferase assay for hetero-dimerization of OsGLK1-N-Luc or OsPSA2-N-Luc with OsPEL1-C-Luc and for homo-dimerization of OsGLK1 and OsPSA2 in their respective N-Luc and C-Luc forms.

In terms of protein localization, OsPEL1 and OsGLK1 were detected in both the nucleus and cytoplasm, whereas OsPSA2 was exclusively localized to chloroplasts (Fig. 6A). Further colocalization analysis confirmed that OsPEL1 and OsGLK1 were both localized in the nucleus and cytoplasm of NT plant protoplasts, as verified by retransfecting OsPEL1-GFP into pOsGLK1:OsGLK1-RFP transgenic plant protoplasts (Fig. 6B). In contrast, no colocalization was observed between OsPEL1 and OsPSA2, indicating that these proteins do not overlap at their final subcellular destinations. Using bimolecular fluorescence complementation (BiFC) analysis, we pinpointed the specific subcellular localization sites where OsPEL1 interacts with OsGLK1, and where it interacts with OsPSA2 (Fig. 6C). As anticipated for the OsGLK1–OsPEL1 interaction, fluorescence signals were observed in both the nucleus and cytoplasm. Interestingly, fluorescence from the OsPSA2–OsPEL1 interaction was also detected in the cytoplasm, accompanied by aggregated forms, in which OsPSA2 did not exhibit its typical localization. Additionally, a split-luciferase assay conducted in planta using tobacco leaves validated these interactions, demonstrating stronger binding between OsPEL1 and OsGLK1, and relatively weaker binding between OsPEL1 and OsPSA2 (Fig. 6D).

**Figure 7.**
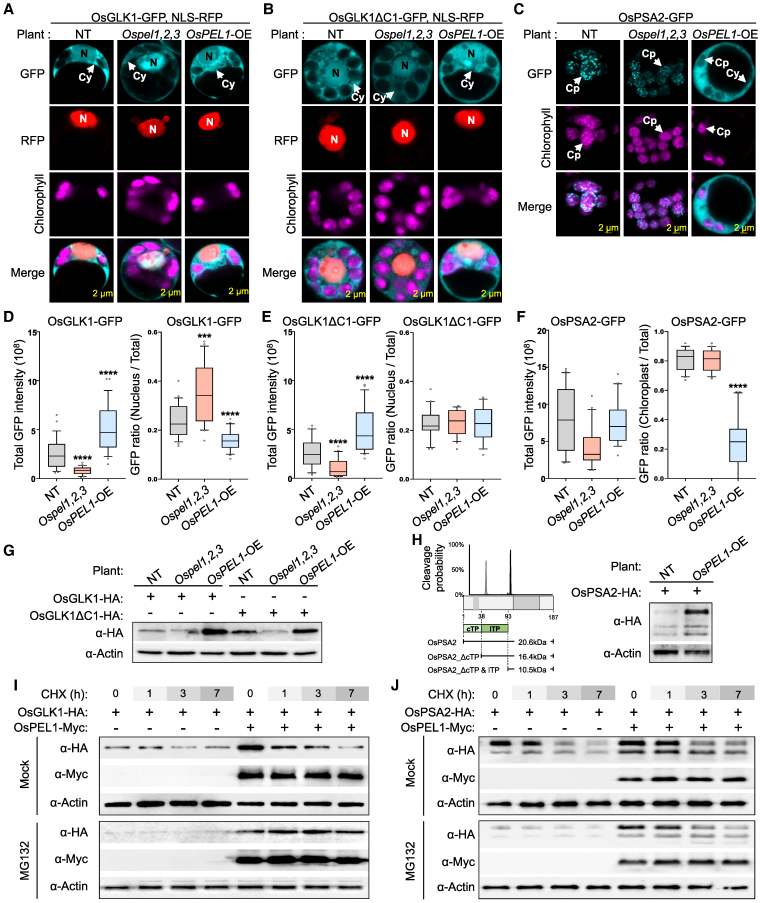
OsPEL1 traps the target proteins in the cytoplasm and increases their protein levels. **A** to **C)** Subcellular localization of OsGLK1 **(A)**, OsGLK1ΔC1 **(B)**, and OsPSA2 **(C)** was examined in rice protoplasts isolated from NT, *Ospel1,2,3*, and *OsPEL1*-OE lines, representing different OsPEL mutants. **D** and **E)** Statistical analysis of GFP fluorescent signals for OsGLK1 **(D)** and OsGLK1ΔC1 **(E)**, calculated as total absolute intensity (left panel) and nucleus-to-total absolute intensity ratio (right panel), with values measured from each cell (*n* = 22). **F)** Statistical analysis of GFP fluorescent signals for OsPSA2, calculated as total absolute intensity (left panel) and chloroplast-to-total absolute intensity ratio (right panel), with values measured from each cell (*n* = 25). Statistical significance in **D** to **F)** is indicated as ***P < 0.001 and ****P < 0.0001 based on Student's *t*-test. And for elements of the boxplots, center line, median; box limits, upper and lower quartiles; whiskers, 10% to 90%; and points, outliers. **G** and **H)** Western blot analysis of protoplasts derived from various background plants transfected with OsGLK1 and OsGLK1ΔC1 **(G)** and OsPSA2 **(H)**, which a schematic of the predicted OsPSA2 cleavage sites based on TargetP 2.0. cTP, chloroplast-targeting transit peptide; lTP, luminal-targeting transit peptide. **I** and **J)** Western blot analysis of NT rice protoplasts cotransfected with OsPEL1 and OsGLK1 **(I)** or OsPSA2 **(J)** following treatment with CHX or a combination of CHX and MH132.

To examine how cytoplasmic interactions mediated by OsPEL1 influence the subcellular localization of target proteins, we analyzed OsGLK1, OsGLK1ΔC1 (a C1 domain-deleted version that does not bind OsPEL1), and OsPSA2 in 3 genetic backgrounds: NT, *Ospel1,2,3*, and *OsPEL1*-OE (Fig. 7). OsGLK1 and OsGLK1ΔC1 exhibited consistent dual localization to the nucleus and cytoplasm across all backgrounds (Fig. 7, A and B). In contrast, OsPSA2, which is typically localized to the chloroplast in NT and *Ospel1,2,3*, was predominantly retained in the cytoplasm in *OsPEL1*-OE plants (Fig. 7C). Quantitative analysis of total GFP fluorescence revealed a general trend of lower intensity in *Ospel1,2,3* (darker green) and higher intensity in *OsPEL1*-OE (paler green) compared to NT (normal green) control (Fig. 7, D to F, left panels). However, subcellular distribution patterns varied: Nuclear OsGLK1 intensity increased by 52% in *Ospel1,2,3* and decreased by 33% in *OsPEL1*-OE relative to NT (Fig. 7D, right panel), suggesting that OsPELs inhibit OsGLK1's nuclear accumulation. In contrast, OsGLK1ΔC1 showed similar nuclear intensities across all genotypes, supporting the idea that the loss of OsPEL1 binding allows unimpeded nuclear localization (Fig. 7E, right panel). For OsPSA2, chloroplast-localized fluorescence remained stable in NT and *Ospel1,2,3* but was markedly reduced in *OsPEL1*-OE, indicating impaired chloroplast targeting (Fig. 7F, right panel). These findings suggest that OsPEL1 sequesters OsGLK1 in the cytoplasm, limiting its nuclear import while also preventing the proper chloroplast localization of OsPSA2 through cytoplasmic retention.

As shown above, the consistently altered total GFP intensity patterns for OsGLK1, OsGLK1ΔC1, and OsPSA2 led us to investigate whether the OsPEL family affects protein abundance, through western blot analyses following the transfection of target constructs into NT, *Ospel1,2,3*, and *OsPEL1*-OE protoplasts (Fig. 7, G and H). Protein levels of OsGLK1 and OsGLK1ΔC1 were decreased in *Ospel1,2,3* and increased in *OsPEL1*-OE relative to NT (Fig. 7G). Similarly, OsPSA2 levels**—**detected as 3 distinct bands representing a premature cytoplasmic form and 2 mature plastidial forms**—**were elevated in *OsPEL1*-OE, with a disproportionately larger increase in the cytoplasmic form compared to NT (Fig. 7H). These findings align with the total GFP data and indicate that OsPEL1 is involved in the transient accumulation of OsGLK1 and OsPSA2, especially in the cytosolic pool of OsPSA2.

To determine whether the observed changes in protein levels are due to altered degradation dynamics, we performed protein stability assays using cycloheximide (CHX) and the proteasome inhibitor MG132 (Fig. 7, I and J). These results further confirm that OsPEL1 is involved in the transient accumulation of OsGLK1 and OsPSA2. Upon CHX treatment, OsGLK1 levels gradually decreased, but this reduction was blocked by MG132 cotreatment, indicating proteasome-mediated degradation (Fig. 7I). This is consistent with prior findings in tomato that showed GLK degradation via a CUL4–DDB1 E3 ligase complex (Tang et al. 2016). On the other hand, OsPSA2 stability depended on subcellular localization. Under CHX treatment with OsPEL1, the cytosolic form degraded rapidly, while the plastid-localized form remained stable, suggesting that OsPEL1 primarily affects the cytosolic pool of OsPSA2, which is more transient and susceptible to degradation. MG132 failed to prevent cytosolic OsPSA2 degradation, suggesting a proteasome-independent pathway (Fig. 7J). Notably, OsPEL1 itself remained stable throughout all treatments, suggesting it is not rapidly turned over (Fig. 7, I and J). Collectively, these findings indicate that OsPEL1 interferes with the subcellular localization of OsGLK1 and OsPSA2 while also affecting their protein accumulation via cytoplasmic retention.

### Loss and gain of function in the OsPEL family reveals transcriptional homeostasis underlying greening-associated gene regulation

**Figure 8.**
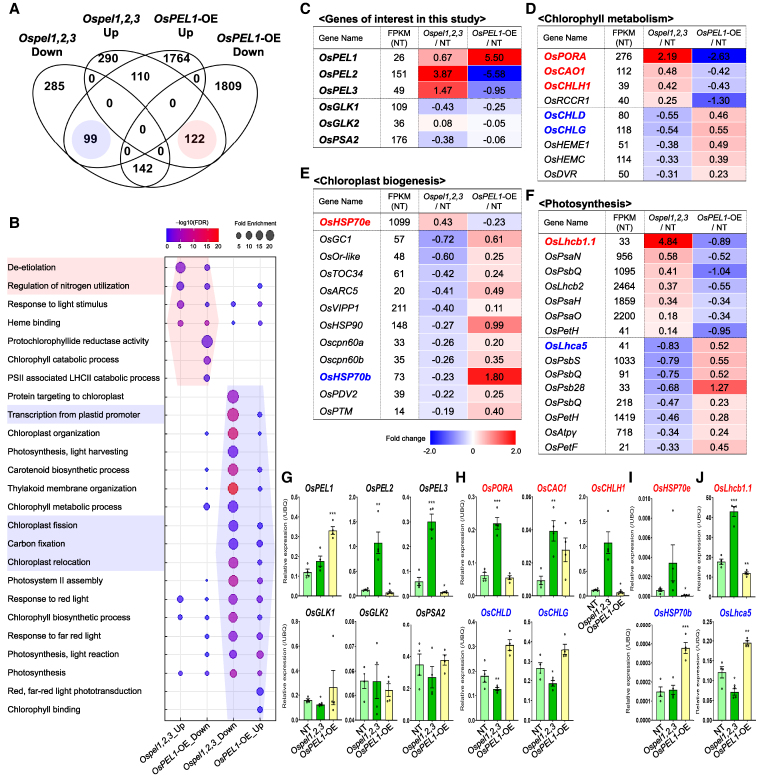
RNA-seq analysis reveals transcriptional homeostasis by *PhANG* expression. **A)** Numbers of DEGs identified in *Ospel1,2,3* and *OsPEL1*-OE compared to NT. Detailed values and lists can be found in Supplementary Data Sets S5 and S6. **B)** GO term enrichment analyses. **C)** Log2 fold change values for the 3 *OsPELs* and its protein–protein binding partners, *OsGLK1*, *OsGLK2*, and *OsPSA2*. **D** to **F)** Log2 fold change values of *PhANGs* involved in chlorophyll metabolism **(D)**, chloroplast biogenesis **(E)**, and photosynthesis **(F)** with boldfaced genes that were used in RT-qPCR. Detailed values and lists can be found in Supplementary Data Set 7. **G)** RT-qPCR validation of the 6 genes shown in **C)**. **H** to **J)** RT-qPCR validation of representative genes: 5 for chlorophyll metabolism **(H)**, 2 for chloroplast biogenesis **(I)**, and 2 for photosynthesis **(J)** using the primers listed in Supplementary Data Set 8. For **G** to **H)**, statistical significance: *P < 0.05, **P < 0.01, ***P < 0.001, and ****P < 0.0001 (Student's *t*-test). And the bars represent the standard error of the mean.

To investigate transcriptomic changes in *Ospel1,2,3* and *OsPEL1*-OE compared to NT plants, we performed RNA-seq analysis and identified differentially expressed genes (DEGs) (Fig. 8A; Supplementary Data Sets S4 to S6). Notably, *OsPEL1*-OE exhibited extensive transcriptomic alterations, with 1,764 upregulated and 1,809 downregulated genes, possibly reflecting a compensatory response to the pale-green phenotype. A key finding was the identification of genes with opposing expression patterns between *Ospel1,2,3* and *OsPEL1*-OE, including 122 genes upregulated in one and downregulated in the other and vice versa for 99 genes (Fig. 8A). Gene Ontology (GO) analysis revealed strong enrichment for terms related to greening traits, including categories where expression patterns were either concordant or contradictory with greening phenotypes (Fig. 8B). Further RNA-seq analysis confirmed *OsPEL1* overexpression in *OsPEL1*-OE plants and upregulation of all 3 *OsPELs* in *Ospel1,2,3*, while *OsPEL2* and *OsPEL3* were downregulated in *OsPEL1*-OE, indicating transcriptional negative-feedback regulation within the *OsPEL* family (Fig. 8C). Transcript levels of known OsPEL1-binding targets, *OsGLK1*, *OsGLK2*, and *OsPSA2*, were not significantly altered in either genetic background (Fig. 8C). We then focused on greening trait-associated genes (listed in Supplementary Data Set 7), selecting representative genes from 3 functional categories: chlorophyll metabolism (Fig. 8D), chloroplast biogenesis (Fig. 8E), and photosynthesis (Fig. 8F). These genes displayed both consistent and opposing expression patterns in relation to the greening phenotypes.

RNA-seq results were validated by RT-qPCR. Expression analysis of 6 target genes (*OsPEL1*, *OsPEL2*, *OsPEL3*, *OsGLK1*, *OsGLK2*, and *OsPSA2*) by RT-qPCR mirrored the RNA-seq results (Fig. 8G). Further RT-qPCR verification was conducted for key green genes involved in chlorophyll metabolism (Fig. 8H), chloroplast biogenesis (Fig. 8I), and photosynthesis (Fig. 8J). Collectively, 5 genes—*OsPORA*, *OsCAO1*, *OsCHLH1*, *OsHSP70e*, and *OsLhcb1.1*—showed a positive correlation with plant greening traits at the transcriptional level, being upregulated in *Ospel1,2,3* and downregulated in *OsPEL1*-OE compared to NT plants. In contrast, 4 genes—*OsCHLD*, *OsCHLG*, *OsHSP70b*, and *OsLhca5—*exhibited a negative correlation, suggesting their involvement in the negative-feedback mechanism underlying plant greening homeostasis.

## Discussion

The *PEL* family, characterized by an evolutionarily conserved A_thal_3526 domain, exhibits conserved functions as negative regulators of chloroplast development and diversified roles across species, as reflected by their various designations: *AtPEL1*/*RPGE2*/*BPG4* in *Arabidopsis* (Ichikawa et al. 2006; Han et al. 2023; Kim et al. 2023a; Tachibana et al. 2024), *Y locus*/*DcRPGE1* in carrot (Iorizzo et al. 2016; Wang et al. 2024), and *DGP1*/*OsPEL1* in rice (Zhang et al. 2021). Phylogenetic analysis suggests that the diversification of the PEL family underscores its significant impact on the adaptation of plants to terrestrial habitats (Tachibana et al. 2024; Fig. 1; Supplementary Data Sets S1 to S3). We infer that the PEL family emerged to regulate the use of excessive light energy, attenuating it in photosynthetic tissues and inhibiting it in specialized nonphotosynthetic tissues, thereby facilitating the early evolutionary transition of photosynthetic organisms to life on land. In this study, we focused on the negative regulatory roles of the PEL family in controlling greening traits in rice. First, individual overexpression of the 3 rice PEL genes led to reduced greening (Fig. 2, C to G; Supplementary Fig. S2). Conversely, complete functional knockout of the family enhanced greening overall (Fig. 2, C to G; Supplementary Fig. S4), ultimately resulting in increased yield (Fig. 2, H to J), improved chloroplast biogenesis (Fig. 3, A to F), elevated ROS scavenging activity (Fig. 3, H and I), and enhanced photosynthetic efficiency (Fig. 3, J and K). We discuss the significance of these findings from the following 3 perspectives.

### Knockout of the PEL family triggers the chloroplast development in BS cells too, representing a potential first step toward C_4_ rice engineering

Corroborating our hypothesis on the necessity of the PEL family to modulate photosynthesis during land colonization—by fine-tuning it in photosynthetic tissues and repressing it in nonphotosynthetic tissues—*OsPEL* gene expression was broadly detected across leaf tissues (Fig. 2A), including photosynthetic parenchyma cells and epidermal cells lacking chloroplasts (Fig. 2B). In rice, a C_3_ plant in which BS cells exhibit limited photosynthetic activity, *OsPEL1* overexpression was more prominent in vascular bundles (nonphotosynthetic tissues) and BS cells, suggesting a role in restricting chloroplast development in these tissues (Supplementary Fig. S1B). Moreover, *OsPEL1* overexpression led to fluorescence collapse in both BS and MS cells in rice, indicating active chloroplast degradation (Fig. 3A; Supplementary Fig. S6). Together, these findings support the idea that the PEL family may have evolved as a regulatory mechanism to limit excessive photosynthetic activity and suppress chloroplast development in BS cells of C₃ species and in nonphotosynthetic tissues, potentially contributing to the adaptation of early land plants.

C_4_ plants offer a significant advantage in photosynthetic efficiency, leading to increased crop productivity (Ferrari and Freschi 2019; Croce et al. 2024). Consequently, the potential for the C_3_-to-C_4_ transition has been emphasized to enhance photosynthetic capacity and overall productivity in crops, leading to the initiation of the C_4_ rice project in 2008 (Ermakova et al. 2020; Furbank et al. 2023). Efforts to engineer C_4_ crops have followed the evolutionary processes of C_4_ photosynthesis, which are categorized into 3 steps: the development of chloroplasts in BS cells (proto-Kranz state), the reduction of chloroplast development in MS cells (C_4_-like state), and the implementation of the enzyme set required for C_4_ photosynthesis (C_4_ state) (Sage et al. 2011, 2014). As a key first step toward engineering C_4_ rice, constitutive overexpression of maize *GLKs* has been shown to induce proto-Kranz anatomy, representing an intermediate state in the evolutionary path from C_3_ to C_4_ (Wang et al. 2017; Ermakova et al. 2020). Two further studies on the heterologous overexpression of maize *GLKs* in rice demonstrated enhanced chloroplast development and improved photosynthetic capacity, accompanied by increases in vegetative biomass and grain yield under field conditions (Li et al. 2020; Yeh et al. 2022). These findings strongly suggest that maize GLKs function as master transcription factors promoting the expression of photosynthesis-related genes and that GLKs in general hold significant potential for applications aimed at improving crop performance (Kim et al. 2023b; Hernández-Verdeja and Lundgren 2024). However, activation of native *OsGLK1* in rice did not result in enhanced green traits in green tissues (Nakamura et al. 2009). This discrepancy has been attributed to distinct expression patterns between maize and rice genes. *ZmGLK1* and *ZmGLK2* functionally specialize in BS and MS cell types (Hall et al. 1998; Yeh et al. 2022), while *OsGLK1* and *OsGLK2* redundantly function in MS cells, with pale-green phenotypes only observed in double knockout mutants (Wang et al. 2013). This raises the possibility that the existence of strong counteracting partners for GLKs represents specialized mechanisms to control excessive photosynthesis. Ubiquitin-mediated degradation has been suggested as one of the regulatory mechanisms for GLKs in tomato and *Arabidopsis* (Tang et al. 2016; Tokumaru et al. 2017). However, this mechanism is yet to be clarified in rice and may not fully account for the regulatory outcomes observed.

Meanwhile, several recent studies have consistently identified PELs as key repressors of GLKs in both rice and *Arabidopsis*, acting through direct protein–protein interactions to inhibit their activity (Zhang et al. 2021; Han et al. 2023; Kim et al. 2023a; Tachibana et al. 2024). As evidenced by the suppressive function of the PEL family on GLKs, our *Ospel1,2,3* rice plants exhibited a similar alteration in leaf anatomy to that previously observed in *ZmG2*-overexpressing rice leaves (Wang et al. 2017; Ermakova et al. 2020), including significantly promoted chloroplast development in both BS cells and MS cells (Fig. 3; Supplementary Fig. S6). With GLKs, at least, released from the influence of PELs, this suggests that the loss of function of PEL family is sufficient to unlock the potential for C_4_ rice, making an initial step toward C_4_ engineering.

### OsPEL1 inhibits nuclear transcription factor and chloroplast-targeted chaperone in the cytoplasm

PELs/RPGEs/BPG4 proteins have been repeatedly identified as binding partners of GLKs in rice and *Arabidopsis* (Zhang et al. 2021; Han et al. 2023; Kim et al. 2023a; Tachibana et al. 2024), with additional interactors including AtMYB4 and DcAPRR2 in *Arabidopsis* and carrot, respectively (Han et al. 2023; Wang et al. 2024). Collectively, these findings strongly support the notion that PELs primarily function as nuclear suppressors of these transcription factors. However, our Y2H screening revealed that OsPEL1 binds to additional targets beyond transcription factors, notably OsPSA2, a molecular chaperone (Supplementary Fig. S7). Direct interactions between OsPEL1 and these 2 target proteins—OsGLK1 and OsPSA2—were individually validated by defining core binding regions (Fig. 4) and through 3D structural modeling, which indicated that OsPEL1 forms stable complexes with both proteins (Fig. 5). Interestingly, these complexes are structurally distinct yet functionally analogous, suggesting a shared mode of repression despite engaging different interaction interfaces (Supplementary Figs. S10 and S11).

Subcellular localization analyses revealed that OsPEL1 overlaps with OsGLK1 in the nucleus and cytoplasm but does not overlap with OsPSA2 in the chloroplasts (Fig. 6, A and B). The binding properties of OsPEL1, including its subcellular localization and interacting affinities, were further validated in rice and tobacco plants (Fig. 6, C and D). Given that both OsPEL1–OsGLK1 and OsPEL1–OsPSA2 interactions were observed in the cytoplasm, and that OsPEL1 exhibits a relatively higher binding affinity for OsGLK1, it is likely that OsPEL1 exerts a more pronounced regulatory effect on OsGLK1 than on OsPSA2. These results support that OsPELs function in the cytoplasm to inhibit subcellular localization, as evidenced by lower localization efficiencies of OsGLK1 in the nucleus and OsPSA2 in the chloroplasts in *OsPEL1*-OE compared to NT and *Ospel1,2,3* plants (Fig. 7, A to F). Furthermore, the protein abundance of OsGLK1 and OsPSA2 was increased in the presence of OsPEL1 and decreased in the absence of the OsPEL family (Fig. 7, G to J), indicating that OsPEL1 promotes their transient accumulation through posttranslational mechanisms, functioning as part of a feedback regulatory system. Protein stability assays revealed that OsGLK1 undergoes proteasome-mediated degradation, whereas cytosolic OsPSA2 is degraded through a proteasome-independent pathway, likely autophagy (Fig. 7, I and J). To further substantiate these findings, direct biochemical validation—such as subcellular fractionation and immunoblotting of endogenous OsGLK1 and OsPSA2—will be required to definitively quantify changes in their subcellular localization.

Collectively, these findings propose a mechanism for the PEL family, which functions as a cytoplasmic inhibitor of 2 positive regulators for greening traits in rice. This mechanism extends the functional scope of the PEL family beyond its previously established role as nuclear suppressors of transcription factors to include cytoplasmic inhibition of both nuclear transcription factors and plastidial chaperone, linking chloroplast biogenesis and PSI assembly.

### The PEL family is a potential genome-editing target for improving photosynthesis and yield in rice

Beyond its well-documented role as negative regulator of greening traits, including chloroplast development and chlorophyll levels, the PEL family has also been identified as suppressor of pigment biosynthesis, such as that of anthocyanins and carotenoids (Iorizzo et al. 2016; Han et al. 2023; Wang et al. 2024). Additionally, it has been suggested to play a role in chloroplast homeostasis through BR signaling, with studies showing increased ROS generation with decreased photosynthetic activity under high-light conditions in the single knockout mutant, *bpg4* (Tachibana et al. 2024). These findings align with our hypothesis that the PEL family may have evolved to modulate excessive photosynthesis when it first emerged in plants, highlighting its role as a homeostatic factor. ROS scavenging activity, assessed by DPPH and ABTS assays, was elevated in *Ospel1,2,3* plants (Fig. 3, H and I). Consistent with this enhanced oxidative stress management, which likely results from improved chloroplast development, *Ospel1,2,3* rice plants also exhibited enhanced photosynthetic efficiency under varying conditions of CO₂ and light (Fig. 3, J and K). These results support the conclusion that rice can effectively manage increased photosynthetic activity through strengthened ROS scavenging capacity associated with chloroplast maturation in the absence of PEL function.

Regarding yield, 2 *Arabidopsis* mutants, *rpge1/rpge2/rpge3* and *pel1/pel2/pel3/pel4*, showed slightly conflicting results: The former exhibited higher seed yield compared to the low seed yield observed in RPGE-OX, while the latter displayed a reduced 1,000 seed weight, albeit without statistical significance (Han et al. 2023; Kim et al. 2023a). In rice, however, our *Ospel1,2,3* plants showed significantly enhanced results in shoot biomass (20%), TGW (31%), and 100GW (5.3%) compared to NT plants (Fig. 2, H to J). Collectively, the differential yield performance between *Arabidopsis* and rice suggests that multiplexed CRISPR-mediated PEL knockout strategies may be more effective in enhancing productivity in cereal crops like rice.

Unexpectedly but consistently, darker green *Ospel1,2,3* showed reduced total GFP intensity (Fig. 7, A to F) and protein levels (Fig. 7, G to J) of target proteins, while paler green *OsPEL1*-OE exhibited higher levels compared to NT plants, suggesting a homeostasis mechanism regulating greening-associated protein levels. RNA-seq data and RT-qPCR validation revealed gene expression patterns that were both consistent and contradictory to the green phenotypes of *Ospel1,2,3* and *OsPEL1*-OE, indicating potential negative-feedback regulation (Fig. 8). Whereas previous PEL studies emphasized the positive correlations in representative photosynthesis-associated nuclear genes (*PhANGs*) expression, such as increased *OsHEMA1*, *OsPORA*, and *OsLHCB4* in *dgp* mutant (Zhang et al. 2021), and repression of *OsHEMA1*, *OsPORA*, *CAO*, *LHCA1*, *LHCA2*, *LHCB3*, and *LHCB4.1* in *RPGE-OX* plants (Kim et al. 2023a).

**Figure 9.**
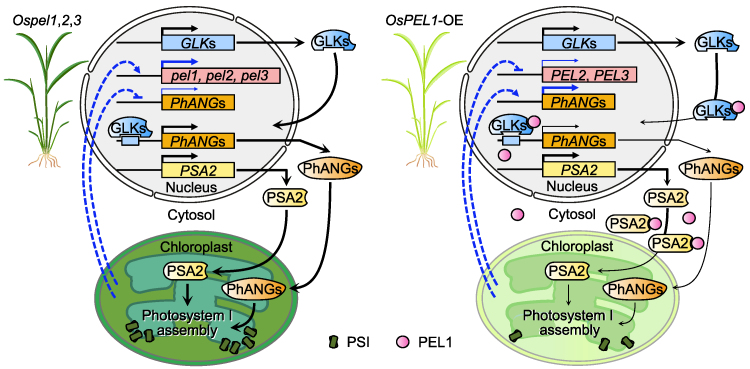
Working model of how the OsPEL family modulates chloroplast development by regulating GLKs and PSA2 in the cytoplasm of rice plants. The *Ospel1,2,3* rice plants displayed darker green phenotypes due to the unrestricted localization of GLKs and PSA2 from PELs to the nucleus and plastids, which led to increased *PhANG* expression and promoted PSI assembly. In contrast, *OsPEL1-*OE rice plants exhibited paler green phenotypes caused by the cytoplasmic sequestration of GLKs and PSA2, resulting in decreased *PhANG* expression and reduced PSI assembly. This model not only explains these localization-dependent effects but also illustrates how chloroplast homeostasis operates at the molecular level through negative-feedback mechanisms (dotted lines), acting to suppress greening in *Ospel1,2,3* plants but enhance greening in *OsPEL1-*OE plants.

Taken together, we propose a model of the OsPEL family in chloroplast development operating in *Ospel1,2,3* and *OsPEL1*-OE rice plants (Fig. 9). The darker green phenotype of *Ospel1,2,3* plants was associated with increased the *PhANG* expression and enhanced PSI assembly, accompanied by reduced OsGLK1 and OsPSA2 protein levels, elevated *OsPEL1, OsPEL2*, and *OsPEL3* transcript levels, and decreased expression of other of *PhANG* sets. In contrast, the paler green phenotype of *OsPEL1*-OE plants resulted from decreased *PhANG* expression and reduced PSI assembly, together with elevated OsGLK1 and OsPSA2 protein levels, reduced *OsPEL2*, and *OsPEL3* transcript levels, and increased expression of other of *PhANG* sets, thus exhibiting opposite trends. These findings suggest that rice regulates greening traits through chloroplast homeostasis mechanisms, involving negative feedback at both protein and transcriptional levels.

In this study, we highlighted the “patroller” function of the PEL family in regulating GLKs and PSA2 in the cytoplasm of rice plants. We identified the PEL family as microProteins, characterized by their small size (78 to 102 amino acids) and strong dominant-negative regulatory functions, like microRNA analogs. Notably, this study reports genome-editing-induced alterations leading to chloroplast-enriched BS cells, resulting in leaf anatomical features uncommon in typical C_3_ plants. These findings suggest that targeted genome editing of the PEL family holds significant potential as a strategy to enhance photosynthesis in rice and other modern crops.

## Materials and methods

### Plant materials and growth conditions

*Japonica*-type Korean rice plants (*Oryza sativa* L. cv. Dongjin) were used as the wild-type control and background cultivar for *Agrobacterium tumefaciens*-mediated transformation (Hiei et al. 1994). These were grown in a growth chamber with a 16-h light/8-h dark cycle at 28 °C during plantlet stages and cultivated in a paddy field at Kyung Hee University in Yongin, Republic of Korea (37° 24′ N latitude, 127° 08′ E longitude).

### Phylogenetic tree construction

Protein sequences of 64 species for a phylogenetic tree were collected from a public database (Phytozome v.12.1.6) (Goodstein et al. 2012) using the *AtPEL* protein sequence as query (Fristedt et al. 2014). The primary results had 235 genes, but the final analysis used 227 genes, excluding 8 genes due to very low similarity. The downloaded protein sequences were aligned by MUSCLE in MEGA11 (https://www.megasoftware.net/; Tamura et al. 2021). A phylogenetic tree was constructed using the maximum likelihood of 1,000 bootstrap in MEGA11.

### Vector construction for the GUS reporter system and histochemical analysis

To generate the *OsPEL1*, *OsPEL2*, and *OsPEL3* promoter:GUS expression vectors, upstream promoter regions of 2,101, 2,006, and 1,884 bp, respectively, along with initial coding sequences of 216, 514, and 182 bp from the start codon of each gene, were amplified by PCR. The resulting PCR products were then cloned into the binary vector *pGA3519* for GUS expression in plants. The primers used for vector construction are listed in Supplementary Data Set 8. Subsequent histochemical GUS staining was performed as previously described (Gho et al. 2017). Briefly, leaf sections from seedling stage plants were hand sliced. The samples were then vacuum infiltrated for 15 min with GUS staining solution and incubated overnight at 37 °C. After chlorophyll removal by washing with 95% ethanol, the samples were photographed using a TM20B microscope (Taeshin-BIOSCIENCE, Namyangju, South Korea).

### RNA isolation, RT-PCR, and RT-qPCR

Rice RNAs were isolated mainly from young leaves and spatiotemporally diverse tissues. Approximately 100 mg of each tissue sample was excised and subjected to RNA isolation using a PureLink Plant RNA Reagent (Invitrogen, Waltham, USA). First-strand cDNAs were generated via reverse transcription reaction with a SuPrimeScript RT Premix (Genet Bio, Daejeon, Republic of Korea). Transcripts of each target gene were detected using RT-qPCR with Prime Q-Master Mix (Genet Bio). The PCR conditions were 1 cycle at 95 °C for 10 min, 40 cycles at 95 °C for 15 s and 58 °C for 15 s, and 1 cycle at 72 °C for 20 s. The relative transcript abundance was calculated using the 2^−ΔΔCT^ method. The numbers are presented as means ± Sd from 4 replicates. The primers used for vector constructions are listed in Supplementary Data Set 8.

### Protoplast isolation

For protoplast isolation, the MSO medium-planted seeds (40 seeds per 1 MSO plate) were grown in dark for 7 d and 1 d of deem light condition (room condition, ∼30 PPFD). After greening, the shoot region of the plant was chopped into pieces 1–2 mm long and immersed in 20 mL enzyme solution [1.5% (w/v) cellulase R-10 (Yakult Pharmaceutical Industry), 0.3% (w/v) macerozyme R-10 (Yakult Pharmaceutical Industry), 0.4 m_D_-mannitol, 2 mm MES, 0.1 × W5 solution, pH 5.7] for 4 h. Then 20 mL W5 solution (154 mm NaCl, 125 mm CaCl_2_, 5 mm KCl, 2 mm MES, pH 5.7) was added to the enzyme solution and all solution was filtered through 40 *μ*m cell strainers (SPL Life Sciences, Pocheon, Republic of Korea). By centrifuging the flow-through at 1000 g for 5 min, the pellet was washed with W5 solution and resuspended with suspension solution (0.4 m mannitol, 20 mm CaCl_2_, 5 mm MES, pH5.7). By using a hemocytometer, 5 × 10^6 cells in 100 *μ*L suspension solution were prepared and mixed with 2 *μ*g of the plasmid. Then, 100 *μ*L of PEG solution (40% PEG4000, 0.4 m mannitol, 100 mm Ca(NO_3_)_2_, pH 5.7) was added, and the mixture was incubated for 20 min. One milliliter of W5 solution was added, and the mixture was centrifuged at 1,000 × *g* for 5 min. After resuspending with 1 mL of WT solution, the mixture was incubated for 12–16 h for expression of target proteins.

### In vitro and in vivo subcellular localization

For a transient expression of GFP fusion protein, full-length cDNAs of *OsPEL1*, *OsPEL2*, and *OsPEL3* were cloned from *O. sativa* L. cv. Dongjin and were individually inserted into a *pGA3651* vector (Kim et al. 2009). Using a PEG-mediated method, each vector was transfected into rice protoplasts that were prepared from shoot tissues of 10-d-old seedlings grown in an MSO medium (He et al. 2016). The primers used for vector constructions are listed in Supplementary Data Set 8.

### Vector construction of overexpression and single and triple CRISPR/Cas9-mediated knockout

For stable overexpression lines of Myc and GFP fusion proteins, full-length cDNAs of *OsPEL* family were cloned from *O. sativa* L. cv. Dongjin and were individually inserted into pGA3427 and pGA3438 vectors, respectively (Kim et al. 2009). Three gRNAs, which target the first exon of *OsPEL1*, *OsPEL2*, and *OsPEL3*, were individually or simultaneously conjugated into *pRGEB32* using the protocol previously described (Xie et al. 2015). The primers used for vector constructions are listed in Supplementary Data Set 8.

### MiniSeq

Mini-sequencing was performed with genome-edited plant genomic DNAs via consecutive triple PCRs, following the manufacturer's instruction (Illumina, San Diego, CA, USA): primary PCRs with a boarder range of target gene regions for nested PCRs, second PCRs with basic adaptor conjugated primers to generate a target gene-specific size of PCR products, and final PCRs with i7 (D701 to D712) and i5 index (D501 to D508)-based adaptor sequences. The index-tagged PCR products were mixed and purified for MiniSeq. The sequencing results were aligned to wild-type genomic sequences using a Cas analyzer (Park et al. 2017). The primers used for primary and secondary PCRs are listed in Supplementary Data Set 8, while those for final PCRs can be found in Illumina Adapter Sequences (https://support.illumina.com/downloads/illumina-adapter-sequences-document-1000000002694.html).

### Chlorophyll measurement

Each 10 mg of fresh flag leaves at 90 days after germination (DAG) was ground with liquid nitrogen, mixed with 1 mL of 100% methanol, and incubated at 70 °C for 30 min with gentle shaking. After being centrifuged at 800 × *g* for 10 min at 4 °C, the absorbance of the supernatant was measured at 653 and 666 nm using the spectrophotometer UV-1800 (SHIMADZU, Kyoto, Japan). Chlorophyll *a* and *b* contents were calculated using an equation from Wellburn's formula as previously (Jeong et al. 2022). The data were analyzed using Student's *t*-test in GraphPad Prism v9 (GraphPad Software, San Diego, CA, USA), and differences were considered significant at *P* < 0.05 (*), *P* < 0.01 (**), *P* < 0.001 (***), or *P* < 0.0001 (****).

### SRM and confocal laser scanning microscopy (CLSM)

To observe the cellular anatomy of rice leaves, a cross-sectional view was prepared using hand-sectioned leaf blades at 14 DAG and obtained from 3D images that were observed from approximately 10-*μ*m-thick surface layers with SRM Elyra7 (ZEISS, Oberkochen, Germany). Further rendering of 3D movies was performed by Vision4D (Arivis, Rostock, Germany). A horizontal view of the same leaf blade was observed by CLSM LSM780 (ZEISS). To observe fluorescence signals in subcellular localization and BiFC analysis from protoplasts and in vivo systems, CLSM LSM780 (ZEISS) was used. Each fluorescence signal was visualized with the following excitation/emission parameters: (i) GFP, AF488 493/517 nm; (ii) RFP, mRF12 590/612 nm; (iii) chlorophyll a, ChloA 655/667 nm; and (iv) calcofluor white, CW2MR 254/432 nm. Cell wall structures were stained with 1% calcofluor white for 10 min and washed thrice with 1 × PBS. Statistical measurements of cell size, chloroplast number, and chlorophyll intensity were analyzed with 30 individual cells per line using an absolute value measurement in ZEN blue edition (ZEISS).

### TEM

Leaf subcellular structures in the middle region of flag leaves were observed by TEM, specifically around the second vein from the main vein of mature plants at 90 DAG. Samples were washed with PBS buffer, postfixed in 2% (w/v) osmium tetroxide (OsO_4_) for 2 h, embedded with epon 812 (Sigma-Aldrich, St. Louis, MO, USA), sectioned with an ultramicrotome EM UC6 (Leica, Wetzlar, Germany), and picked up by formvar-coated copper grids. After poststaining with 2% uranyl acetate for 10 min and lead citrate for 2 min, the specimens were viewed under a Tecnai G^2^ 20 S-TWIN TEM (FEI Company, Hillsboro, OR, USA). MS cells were carefully selected from the first cell layer next to BS cells for consistency.

### ROS scavenging antioxidant activity

The DPPH and ABTS radical-scavenging activities of the extracts were determined using a previously described method (Yang et al. 2018). For DPPH activity, a 0.1 mm DPPH solution in 80% methanol (v/v) was adjusted to an absorbance value of 0.65 ± 0.03 at 517 nm. Then, 0.05 mL of the sample (1 mg/mL) was added to 2.95 mL of the DPPH solution. The absorbance was measured at 517 nm after a 30-min incubation at room temperature in a dark chamber. For ABTS activity, 2.5 mm ABTS and 1 mm 2,2′-azobis (2-amidinopropane) dihydrochloride in phosphate-buffered saline were mixed and heated at 70 °C for 40 min. The ABTS radical solution (0.98 mL), filtered through a 0.45 *μ*m syringe filter, was added to 0.02 mL of the sample (1 mg/mL). The absorbance was measured at 734 nm after a 10-min incubation at 37 °C. DPPH and ABTS radical-scavenging activities were expressed as milligrams of vitamin C equivalents (VCE) per gram dry weight (DW).

### Photosynthesis and agronomic trait measurement

The net photosynthetic rate was recorded by measuring gas exchange parameters in the middle region of flag leaves under various light and CO_2_ conditions using a portable photosynthesis system (Li-6400XT; LI-COR, Lincoln, NE, USA). In detail, the flow rate was set to 500 *μ*mol s^−1^, while the fan speed was set to “fast.” The temperature was maintained at 28 °C, while the leaf surface was measured. To evaluate their agronomic performance, plants of T3 homozygous lines were grown in a paddy field, and their yield parameters were scored as previously (Jeong et al. 2022). The panicle length (cm) and culm length (cm) were measured when the seeds were fully ripened. The filling rate (%), TGW (g), 100GW (g), total number of grains (g), number of panicles per plant, and number of spikelets per panicle were measured after the grains were harvested, threshed, and totally dried. The number of grains was counted with Countmate MC1000H (Motex Ltd., Seoul, Korea). The shoot biomass was measured by using fully dried shoot from 50 pot-tray-grown DAG 50 plants. The data were analyzed using Student's *t*-test in GraphPad Prism v9 (GraphPad Software), and differences were considered significant at *P* < 0.05 (*), *P* < 0.01 (**), *P* < 0.001 (***), or *P* < 0.0001 (****).

### Y2H screening

The Y2H screening has been performed by the company PANBIONET (PANBIONET Corp, Pohang, South Korea; http://www.panbionet.com). In brief, full lengths of OsPEL1 were used as bait from rice leaf cDNA library yPO0803A within AH109 yeast cell line.

### Y2H

The full lengths of 3 OsPELs and partial forms of OsPEL1 were cloned to have a DNA-binding domain into a pGBT9 vector (Clontech, Mountain View, CA, USA), and the full lengths of 2 OsGLKs and partial forms of OsGLK1 were cloned to have an activation domain into the *pGAD424* vector (Clontech). The yeast cell line YRG2 was used for transformation using a small-scale LiAc procedure from Yeast Protocols Handbook (Clontech). To verify the cotransformation, a double dropout medium (SD/-Leu-Trp) was used to select 3 biologically replicate colonies. To test the interactions, a triple dropout medium (SD/-Leu-Trp-His) was used with a double dropout medium (SD/-Leu-Trp) as a control. Each drop contains 13 *μ*L of OD^600^ 0.05 cells, which was diluted by sterilized water from freshly grown cells until OD^600^ 0.4 was obtained in the double dropout medium (SD/-Leu-Trp). The primers used for vector constructions are listed in Supplementary Data Set 8.

### Building in silico 3D structure

The 3D structures of target proteins were predicted by using AlphaFold (Jumper et al. 2021) with the support of molecular visualization program UCSF ChimeraX (Meng et al. 2023). To build protein complex, OsPEL1, OsPEL2, OsPEL3, OsGLK1, OsPSA2, OsGLK1 with OsPEL1, and OsPSA2 with OsPEL1 a.a. sequences were used as input sequences for AlphaFold. For structural reliability, the 3 indicators, Local Distance Difference Test (lDDT), Many-against-Many sequence searching (MMseq2), and PAE were shown for every 3D modeling. To elucidate specific a.a. for protein binding, intersection of PAE data and alanine scanning by using mCSM-PPI2 (Rodrigues et al. 2019) has been selected with threshold default and 1.0 ΔΔG affinity (kcal/mol), respectively. The selected a.a. has been shown for blue (for OsPEL family) or red (for target proteins) in 3D model. The pseudobonds of 3D models showed “contact” value from PAE values by using AlphaFold (Jumper et al. 2021).

### BiFC

For BiFC experiment, *pSY736YN* and *pSY735YC* vectors containing either N- or C-terminal half of the YFP were used (Bracha-Drori et al. 2004). The full-length OsGLK1 was cloned into *pSY736YN* and *pSY735YC*, and the full lengths of OsPEL1, OsPEL2, and OsPEL3 were individually cloned into *pSY735YC*. The nucleus marker vector contained an RFP protein, which was fused with nuclear localization signal peptide “PKKKRKV” (Adam et al. 1989). The primers used for vector constructions are listed in Supplementary Data Set 8.

### Split-luciferase analysis

Target proteins are cloned with *pGWB-cLUC* and *pGWB-nLUC* vectors for C-term and N-term luciferase fusion protein expression. Then, they are infiltrated into tobacco leaf through p19 gene included GV3101 agrobacterium. The infiltrated leaf was dark treated for 1 d and recovered for 3 d before observation. The transformed leaves were infiltrated with 2 mm luciferin and observed with Alliance LD4 gel imaging system (UVItec Ltd.). The primers used for vector constructions are listed in Supplementary Data Set 8.

### Western blot

For Western blot, the protoplast was collected by 1000 × *g* centrifuge for 5 min after 16 h of plasmid transfection. The pellets are vortexed with 60 *μ*L of RIPA buffer [with 1 × Protease Inhibitor Cocktail (Sigma-Aldrich)] and mixed with sample buffer [Laemmli's SDS (GenDEPOT, Katy, TX, USA)]. After boiling for 10 min and centrifuging by 13,400 × *g* for 10 min, the supernatants are loaded into 15% SDS-PAGE gel. The gels were transferred to 0.2 *μ*m PVDF membrane (Cytiva, Marlborough, MA, USA) and detected for target antibodies. For detection of common antibody, such as Myc, HA, and actin, Rabbit anti-Myc Tag Antibody A190-105A (Bethyl, Montgomery, TX, USA), Rabbit anti-HA Tag Antibody A190-108A (Bethyl), and Rabbit anti-Actin antibody CSB-PA000352 (CUSABIO, Houston, TX, USA) have been used. For secondary antibody, HRP-conjugated anti-rabbit IgG (Promega, Madison, WI, USA) is used. After antibody treatment, ECL solution (GenDEPOT) was treated, and images were captured by Alliance LD4 gel imaging system (UVItec Ltd., Cambridge, UK). The primers used for vector constructions are listed in Supplementary Data Set 8.

### Prediction of cleavage site in OsPSA2

The full-length a.a. sequence of OsPSA2 was used to predict cleavage site by TargetP 2.0 program (Armenteros et al. 2019). The result suggested 2 picks of cleavage site and indicates 2 separated transit peptide (TP) signals. The first TP indicates chloroplast targeting transit peptide (cTP) and second TP indicates lumenal targeting transit peptide (lTP) (Yu et al. 2023).

### RNA-seq

Total RNA samples (RIN > 7.3) were prepared using the middle region of rice flag leaves at 90 DAG for NT, *Ospel1,2,3*, and *OsPEL1*-OE plants with biological triplicates. Transcriptome data were achieved by an Illumina platform with paired-end sequencing in a NovaSeq 6000 sequencing system (Illumina). In each transcriptome sample, 100 bp paired-end sequences were assessed using FastQC (Supplementary Data Set 4). Any adapter contaminations or low-quality sequences (-q 30) were removed using both fastp (Chen et al. 2018) and its wrapper tool, Trim Galore. Read pairs were aligned to the rice genome (International Rice Genome Sequencing Project 1.0 reference genome) using TopHat2, and read counts for each gene and DEGs were evaluated using Cuffdiff (Ghosh and Chan 2016). Genes with *P*-value of <0.05 and log2 fold changes of >1 based on fragments per kilobase per million fragments mapped (FPKM) values were considered as DEGs. The GO term was classified by following a ShinyGO 0.80 database (Ge et al. 2020). The false discovery rate (Benjamini–Hochberg correction) was used for ranking GO enrichment analysis.

### Statistical analysis

Statistical analysis methods were used as indicated in the figure legends. Student's *t*-test was performed with Excel (version 16.0).

### Accession numbers

Sequence data from this article can be found in the GenBank/EMBL data libraries under accession numbers BGIOSGA004763 (*OsPEL1*, LOC_Os01g62060), BGIOSGA012351 (*OsPEL2*, LOC_Os03g17200), BGIOSGA017909 (*OsPEL3*, LOC_Os05g38680), BGIOSGA022875 (*OsGLK1*, LOC_Os06g24070), BGIOSGA002040 (*OsGLK2*, LOC_Os01g13740), and BGIOSGA028803 (*OsPSA2*, LOC_Os08g36140).

## Supplementary Material

koaf235_Supplementary_Data

## Data Availability

The data underlying this article are available in the article and in its online supplementary material.

## Supplementary data

The following materials are available in the online version of this article.

**Supplementary Figure S1.** Dual subcellular localization of the OsPEL family in nuclei and cytoplasm (supports Fig. 2).

**Supplementary Figure S2.** Overexpression of the *OsPEL* family shows a paler green phenotype and reduced chlorophyll accumulation (supports Fig. 2).

**Supplementary Figure S3.** Single CRISPR lines individually constructed for 3 *OsPEL* genes (supports Fig. 2).

**Supplementary Figure S4.** Triple CRISPR lines and backcrossing analysis to confirm redundancy of OsPEL family (supports Fig. 2).

**Supplementary Figure S5.** Six agronomic traits with no significant changes in *Ospel1,2,3* line compared to NT and *OsPEL1*-OE plants (supports Fig. 2).

**Supplementary Figure S6.** Single-channel results from Fig. 3A show increased chloroplast development in the *Ospel1,2,3* plants compared to NT and *OsPEL1*-OE plants (supports Fig. 3).

**Supplementary Figure S7.** Y2H screening results using OsPEL1 as bait identified OsGLK1, OsGLK2, and OsPSA2 as OsPEL1-binding candidates (supports Fig. 4).

**Supplementary Figure S8.** 3D structure modeling of the OsPEL family (supports Fig. 5).

**Supplementary Figure S9.** 3D structure modeling of OsGLK1 and OsPSA2 (supports Fig. 5).

**Supplementary Figure S10.** Detailed information of 3D modeling from OsPEL1 and OsGLK1 complex shown in Fig. 5A.

**Supplementary Figure S11.** Detailed information of 3D modeling from OsPEL1 and OsPSA2 complex shown in Fig. 5B.

**Supplementary Data Set 1.** Total gene lists of the PEL family in 64 photosynthetic plant species (supports Fig. 1).

**Supplementary Data Set 2.** FASTA (.txt) file of the alignment in Fig. 1.

**Supplementary Data Set 3.** Newick (.nwk) file for phylogeny in Fig. 1.

**Supplementary Data Set 4.** Quality control (QC) results of RNA-seq analysis (supports Fig. 8).

**Supplementary Data Set 5.** Significantly altered genes in the *Ospel1,2,3* compared to NT (supports Fig. 8).

**Supplementary Data Set 6.** Significantly altered genes in *OsPEL1*-OE compared to NT (supports Fig. 8).

**Supplementary Data Set 7.** Detailed fold change values of *PhANGs* and *PhAPGs* in Fig. 8.

**Supplementary Data Set 8.** List of primers used in this study (supports Fig. 2, 4, and 6 to 8).
