# Supplementary material for: Editing of rice PSEUDO-ETIOLATION IN LIGHT microProtein genes promotes chloroplast development
Source: Plant Cell. 2025 Oct 7;37(11):koaf235. doi: 10.1093/plcell/koaf235 (PMC12696372; doi:10.1093/plcell/koaf235)
Supplement: koaf235_Supplementary_Data [file koaf235_Supplementary_Data.zip › TPC-2025-0564R2_EDITED-HB2_Supp-mod2-no track.pdf]

Supplementary Information for

Editing of rice *PSEUDO-ETIOLATION IN LIGHT* microProtein genes promotes chloroplast development

Heebak Choi, Tae Gyu Yi, Yun-Shil Gho, Ki-Hong Jung, Sun-Hwa Ha

Corresponding author: Email: sunhwa@khu.ac.kr

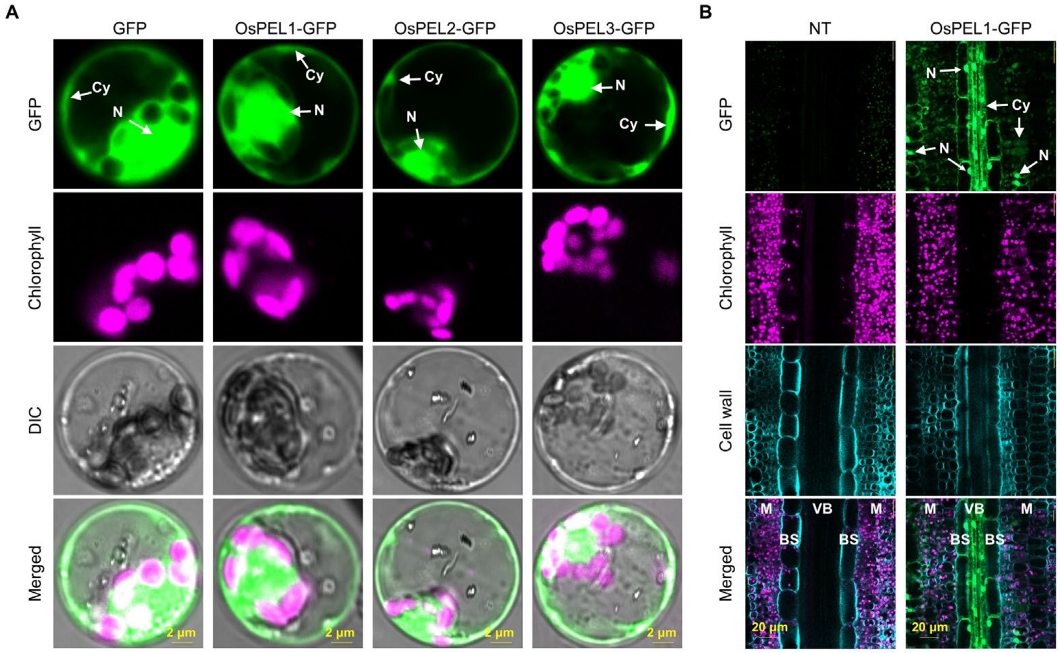

Supplementary Figure S1. Dual subcellular localization of the OsPEL family in nuclei and cytoplasm (Supports Figure 2). **A)** GFP fluorescence showing the subcellular localization of the OsPEL family in transiently transfected rice protoplasts. White arrows point to the nucleus (N) and cytoplasm (Cy). **B)** GFP fluorescence depicting the subcellular localization of OsPEL1 in the vertical view of stably transformed rice leaves. Purple represents the autofluorescence signal of chlorophylls, while cyan indicates the cell wall structure stained with calcofluor white. Bundle sheath cells (BS), mesophyll cells (M), and vascular bundle (VB) are labeled in the corresponding regions.

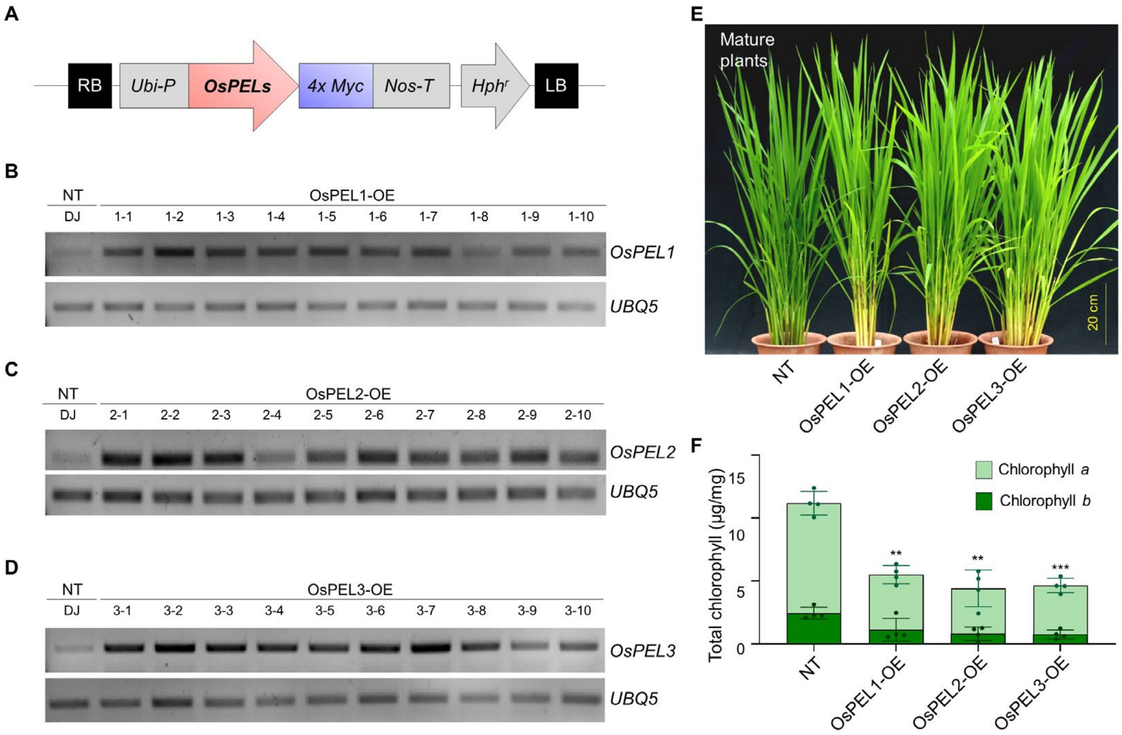

Supplementary Figure S2. Overexpression of the *OsPEL* family shows a paler green phenotype and reduced chlorophyll accumulation (Supports Figure 2). **A)** Schematic diagram of overexpression vectors for *OsPEL* family genes using *pGA3438* as a backbone vector. **B** to **D)** RT-PCR analysis of *OsPEL1* **(B)**, *OsPEL2* **(C)**, and *OsPEL3* **(D)** to verify their overexpression in T0 plants. **E)** Plant phenotypes of representative overexpression lines for three *OsPEL* genes at 90 DAG. **F)** Chlorophyll contents of representative overexpression lines for three *OsPEL* genes at 90 DAG (n = 4, the bars represent the standard error of the mean, ***P* < 0.01 and ****P* < 0.001, Student's t test).

A

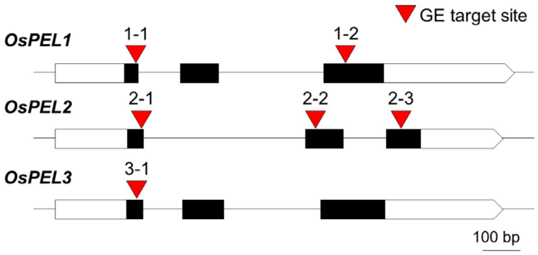

B

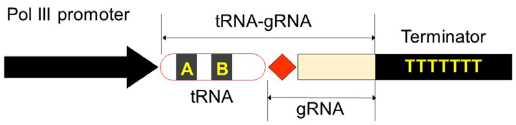

C

Gene (Target site)	Regenerated Plant No.	Sequenced Plant No.	Mutated Plant No.	Bi-allelic hom o plant No.
OsPEL1 (1-1)	>44	20	2 (10%)	0
OsPEL1 (1-2)	>32	8	7 (87.5%)	4 (50%)
OsPEL2 (2-1)	>24	12	12(100%)	7(58%)
OsPEL2 (2-2)	84	20	18 (90%)	15 (75%)
OsPEL2 (2-3)	24	20	9 (45%)	0 (0%)
OsPEL3 (3-1)	>56	20	3 (15%)	0

D

OsPEL1

```
(1-1)     WT CGTCGCCGGCT TCGTACATCAGATTGGTACGTC
      #73_3 CGTCGCCGGCT TCGTACATCAGATTGGTACGTC
            CGTCGCCG---- --------------GTACGTC   -18bp
      #74_2 CGTCGCCGGCT TCGTACATCAGATTGGTACGTC
            CGTCGCCGGCTtTCGTACATCAGATTGGTACGTC   +1bp

(1-2)     WT CTTCGCCACGT ACAAGAAGGGCCAAGGAGAGGA
      #4-04 CTTCGCCACGTaACAAGAAGGGCCAAGGAGAGGA   +1bp
            CTTCGCCACGTaACAAGAAGGGCCAAGGAGAGGA   +1bp
      #4-08 CTTCGCCACGT -----AAGGGCCAAGGAGAGGA   -5bp
            CTTCGCCACGT ----GAAGGGCCAAGGAGAGGA   -4bp
```

OsPEL2

```
(2-1)     WT GACTCCCCTGC CTCTTACATCCACATGGTAATC
      #5-23 GACTCCCCTGC C------ATCCACATGGTAATC   -6bp
            GACTCCCaTGCcCTCTTACATCCACATGGTAATC   +1bp
      #5-24 GACTCCCCTGCtCTCTTACATCCACATGGTAATC   +1bp
            GACTCCCaTGC--------(30bp)---------   -30bp

(2-2)     WT CCTGATAGAGAAATGCATGACG TTCGGGATGAG
      #27_7 CCTGATAGAGAAATGCATGAC- TTCGGGATGAG   -1bp
            CCTGATAGAGAAATGCATGACGcTTCGGGATGAG   +1bp
      #30_5 CCTGATAGAGAAATGCATGACGtTTCGGGATGAG   +1bp
            CCTGATAGAGAAATGCATGACGtTTCGGGATGAG   +1bp

(2-3)     WT AAGTACAAGCAGTTGAGATCAG  AAAAGGGAGGC
      #36_6 AAGTACAAGCAGTTGAGATCAGatAAAAGGGAGGC  +2bp
            AAGTACAAGCAGTTGAGA---a  AAAAGGGAGGC  -3bp
      #39_5 AAGTACAAGCAGTTGAGAT---  AAAAGGGAGGC  -3bp
            AAGTACAAGCAGTTGAGAT---  -AAAGGGAGGC  -4bp
```

OsPEL3

```
(3-1)     WT TCATCCTCAGCTTCGTACATCA GAATGGTATAT
      #67_3 TCATCCTCAGCTTCGTACATCA GAATGGTATAT
            TCATCCTCAGCTTCGTA----- ---TGGTATAT   -8bp
      #68_2 TCATCCTCAGCTTCGTACATCA GAATGGTATAT
            TCATCCTCAGCTTCGTAC---- GAATGGTATAT   -4bp
```

Sky blue, PAM sequence; Red, changed sequence;
Bold, target sequence

Supplementary Figure S3. Single CRISPR lines individually constructed for three *OsPEL* genes (Supports Figure 2). **A)** Location of gRNA target sites for each *OsPEL* gene. **B)** Schematic diagram of a single CRISPR vector used for genome editing. **C)** Summary of genome-editing frequency for each single CRISPR line in T0 plants. **D)** Representative genome sequences of target sites of three *OsPEL* genes in T0 plants.

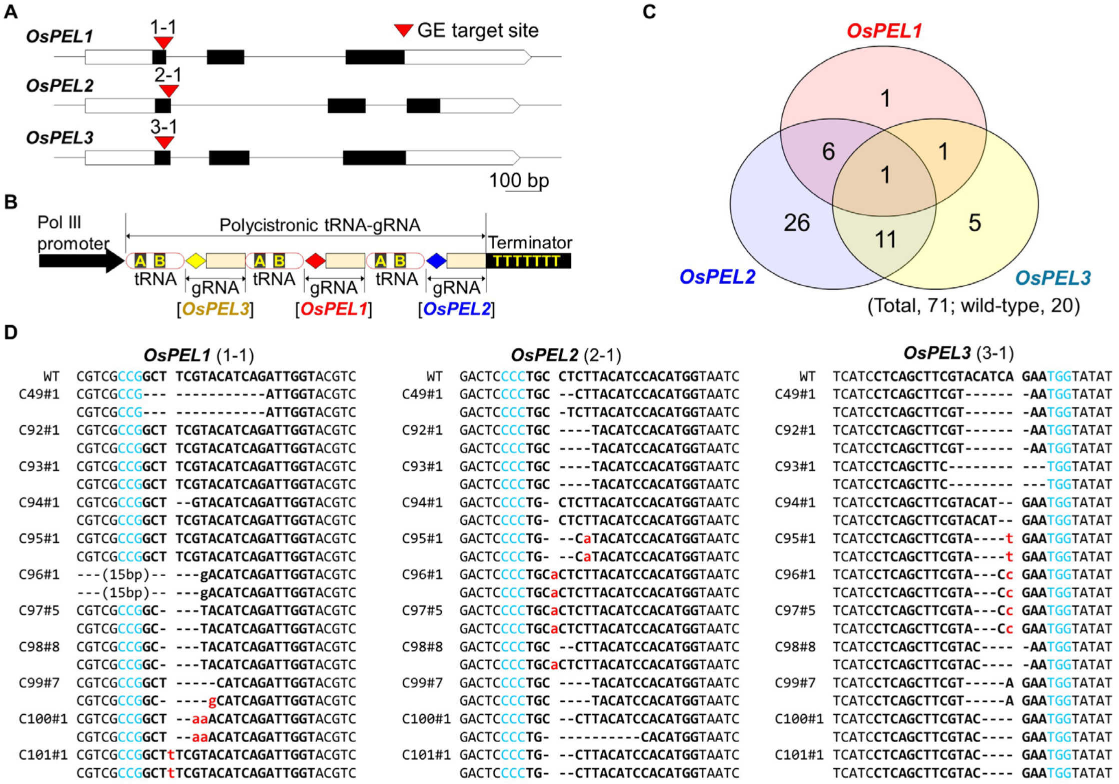

Sky blue, PAM sequence; Red, changed sequence; **Bold**, target sequence

(Continued)

E

Target gene / Plant	Genotyping result *OsPEL1*	*OsPEL2*	*OsPEL3*
WT	wt	wt	wt
**C49#1**	**-14**	**-2/-1**	**-7**
C92#1	wt	-4	-7
C93#1	wt	-4	-11
C94#1	wt/-2	-1	-2
C95#1	wt	-1	-2
C96#1	-15	+1	-3
C97#5	-4	+1	-3
C98#8	-4	-2/+1	-5
C99#7	-5/-5	-4	-5
C100#1	-2	-2/-11	-4
C101#1	+1	-3	-4

*Gray lines indicate triple knockout lines.
*Bold line indicates the line used for backcrossing.

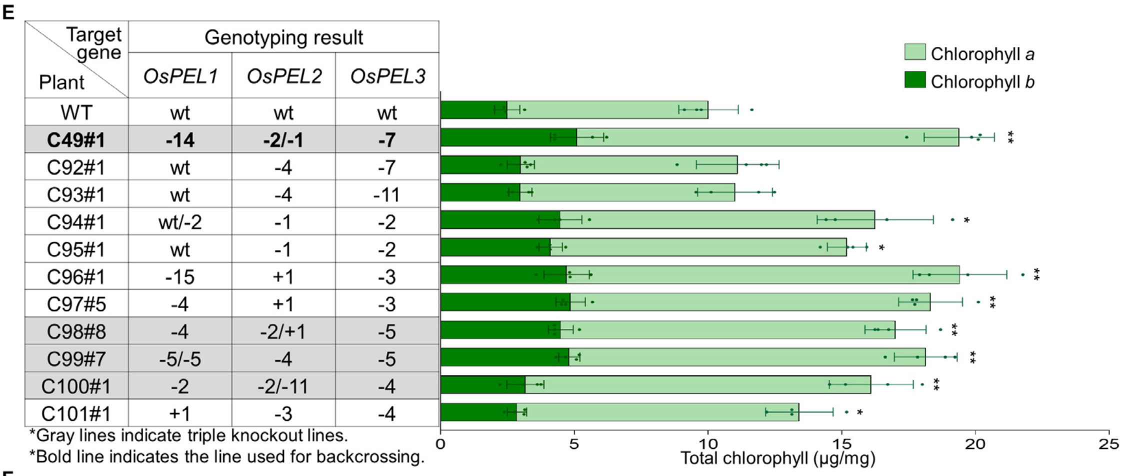

F

Target gene / Plant	Genotyping result *OsPEL1*	*OsPEL2*	*OsPEL3*
WT	wt	wt	wt
BC_92#2	wt	wt	wt
BC_25#1	wt	-2	wt
BC_95#3	wt	-2	wt
BC_26#1	wt	wt	-7
BC_92#1	wt	wt	-7
BC_28#1	-14	wt	-7
BC_29#1	wt	-2	-7
BC_47#1	-14	-2	-7
BC_109#1	-14	-2	-7
BC_120#1	-14	-2	-7

*Gray lines indicate triple knockout lines.

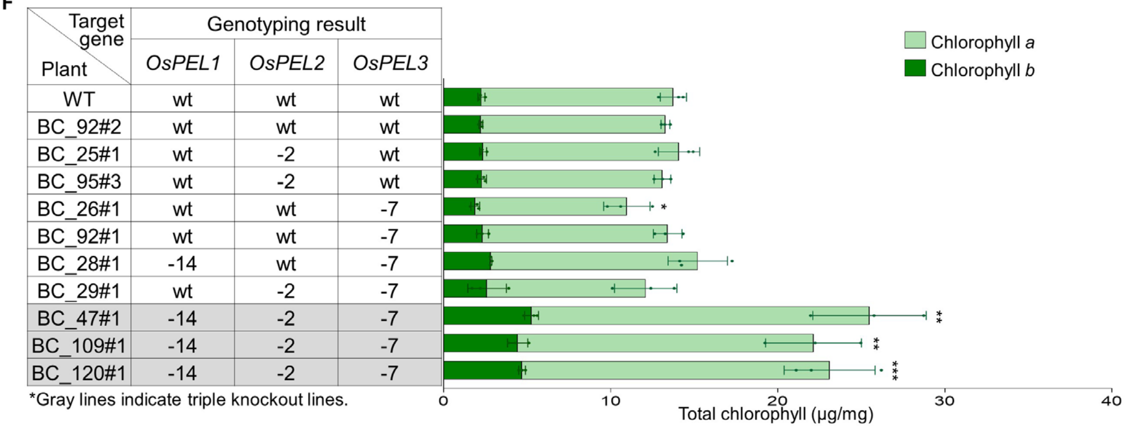

Supplementary Figure S4. Triple CRISPR lines and backcrossing analysis to confirm redundancy of OsPEL family (Supports Figure 2). **A)** Location of gRNA target sites for multiple CRISPR editing of *OsPEL* genes. **B)** Schematic diagram of the triple CRISPR vectors used for genome editing. **C)** Venn diagram showing the number of biallelic homozygous plants from hygromycin-selected 71 T0 plants. **D)** Genome-edited sequences analyzed by MiniSeq in representative lines with triple knockouts. **E)** Genotyping results for three *OsPEL* genes along with their corresponding chlorophyll contents (n = 4, the bars represent the standard error of the mean). **F)** Backcrossing results of the C49#1 progeny for segregated OsPEL family mutations and their related chlorophyll contents (n = 4, the bars represent the standard error of the mean, $*P < 0.05$, $**P < 0.001$ and $***P < 0.001$, Student's *t*-test).

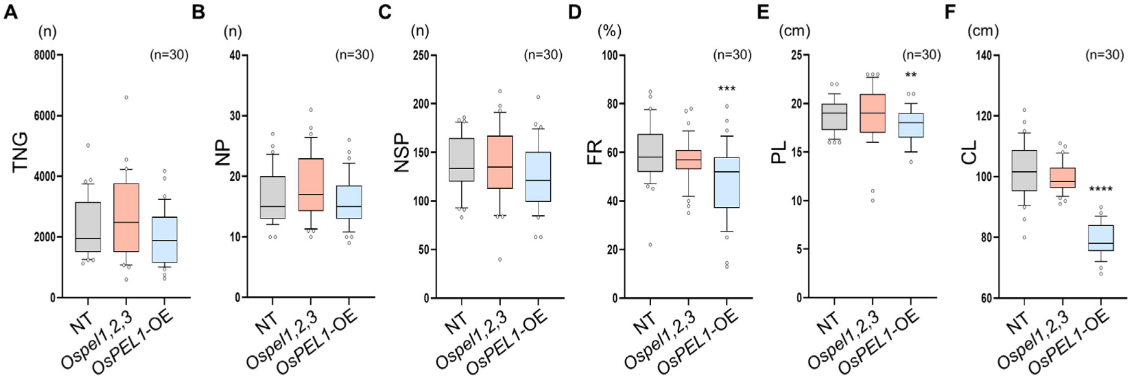

Supplementary Figure S5. Six agronomic traits with no significant changes in *Ospel1,2,3* line compared to NT and *OsPEL1*-OE plants (Supports Figure 2). **A** to **F)** The agronomic traits of the total number of grains (TNG) **(A)**, number of panicles per plant (NP) **(B)**, number of spikelets per panicle (NSP) **(C)**, filing rate (FR) **(D)**, panicle length (PL) **(E)**, and culm length (CL) **(F)** measured with field-grown T3 plants (n = 30, **P < 0.01, ***P < 0.001 and ****P < 0.0001, Student's *t*-test). For elements of the boxplots, center line, median; box limits, upper and lower quartiles; whiskers, 10 to 90 %; points, outliers.

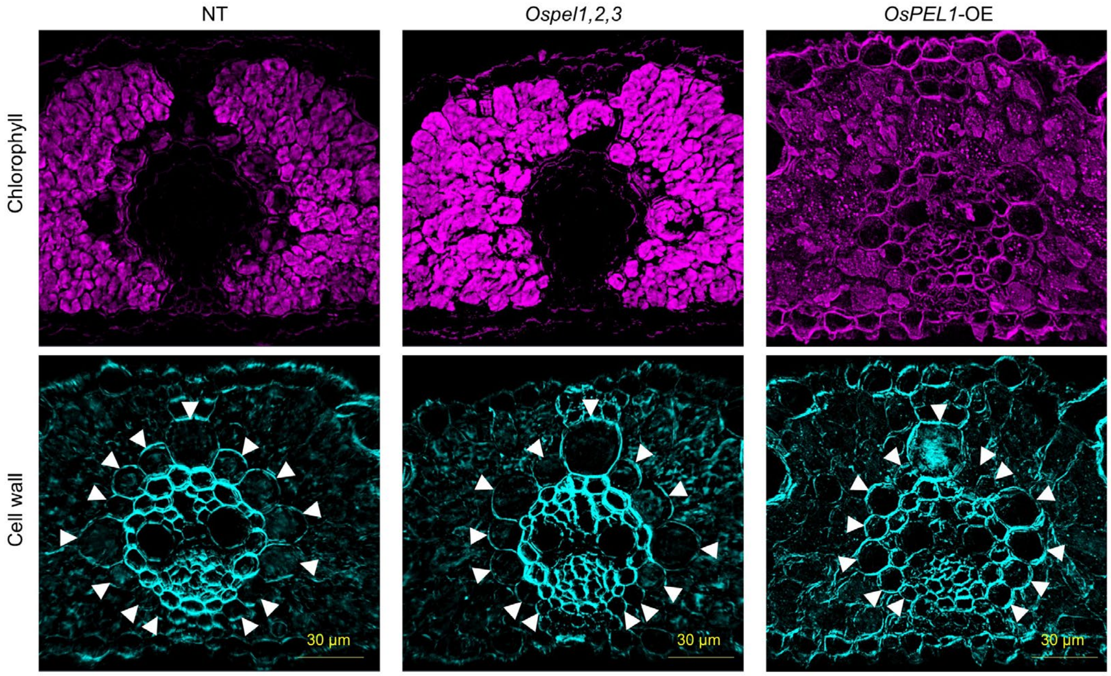

Supplementary Figure S6. Single-channel results from Fig. 3A show increased chloroplast development in the *Ospel1,2,3* plants compared to NT and *OsPEL1*-OE plants (Supports Figure 3). Chlorophyll (in pink) and cell wall (in cyan) were observed by auto-florescence and calcofluor white staining, respectively. BS cells are marked with white arrow heads.

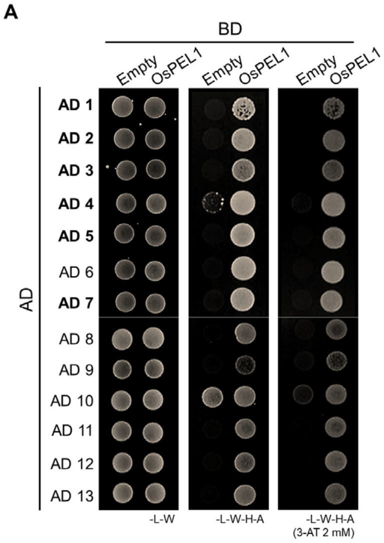

B

Prey ID	Locus ID	Gene Name	Protein size and position (a.a.)		
			F	N	C
**AD 1**	**LOC_Os06g24070**	**GOLDEN2-LIKE 1 (OsGLK1)**	**455**	1-149	**306-455**
**AD 2**	**LOC_Os08g36140**	**PHOTOSYSTEM I ASSEMBLY 2 (OsPSA2)**	**187**	1-97	98-187
**AD 3**	**LOC_Os01g13740**	**GOLDEN2-LIKE 2 (OsGLK2)**	**539**	n.t.	n.t.
**AD 4**	**LOC_Os03g58330**	**Transcription factor UNE12 (OsbHLH098)**	**294**	1-129	130-294
**AD 5**	**LOC_Os06g24070**	**GOLDEN2-LIKE 1 (OsGLK1)***	**455**	1-149	**306-455**
AD 6	LOC_Os02g36619	EXO70FX6*	700	n.t.	n.t.
**AD 7**	**LOC_Os07g01140**	**BTB domain with MATH domain (OsMB9)**	**395**	1-155	**156-395**
AD 8	LOC_Os01g69950	50S Ribosomal protein L27 (OsRP_L27)	195	n.t.	n.t.
AD 9	LOC_Os09g32810	Ribulose 5-phosphate 3-epimerase (OsRPE)	205	n.t.	n.t.
AD 10	LOC_Os12g44110	NRT1/ PTR FAMILY 2.11 (OsNRT)	587	n.t.	n.t.
AD 11	LOC_Os12g44110	NRT1/ PTR FAMILY 2.11 (OsNRT)	587	n.t.	n.t.
AD 12	LOC_Os06g48780	60S acidic ribosomal protein P3-like	119	n.t.	n.t.
AD 13	LOC_Os12g22760	Expressed protein*	102	n.t.	n.t.

* The target sequences are inserted in off-frame; n.t. not tested; gray cell indicates positive binding.

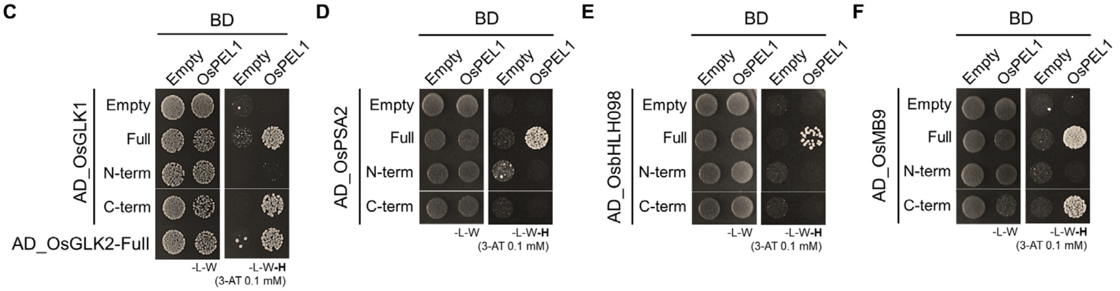

Supplementary Figure S7. Y2H screening results using OsPEL1 as bait identified OsGLK1, OsGLK2 and OsPSA2 as OsPEL1 binding candidates (Supports Figure 4). **A)** Thirteen positive colony were selected and validated for candidate identification. **B)** List of 13 positive colonies with MSU locus and putative functions. **C** to **F)** OsPEL1 binding analysis through fragmentation of target proteins, OsGLK1/OsGLK2 **(C)**, OsPSA2 **(D)**, OsbHLH098 **(E)** and OsMB9 **(F)**. The result of AD_OsGLK1 and BD in (C) is a collage of Fig. 4B. The binding results for (C) to (F) are stated in gray cells of (B).

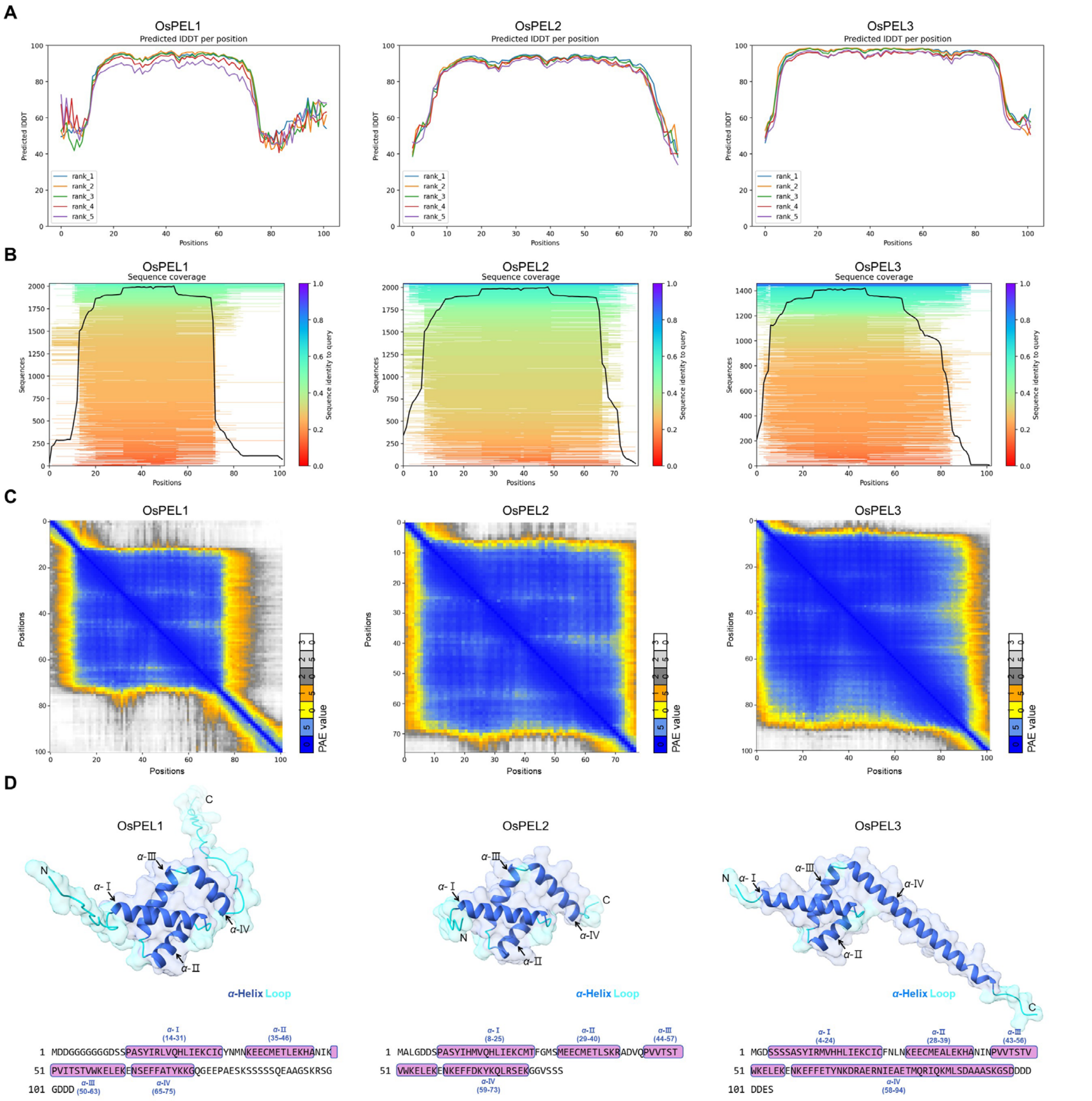

Supplementary Figure S8. 3D structure modeling of the OsPEL family (Supports Figure 5). **A** to **C)** The structural reliability and expected position error among OsPEL family proteins were analyzed by Local Distance Difference Test (lDDT) **(A)**, Many-against-Many sequence searching (MMseqs2) **(B)** and Predicted Aligned Error (PAE) plot **(C)**. The lDDT includes five top ranking structures and ranking #1 construct is used for further analysis. **D)** The conserved four α-helix structures of OsPEL family were shown with 3D modeling (up) and amino acid sequences (down).

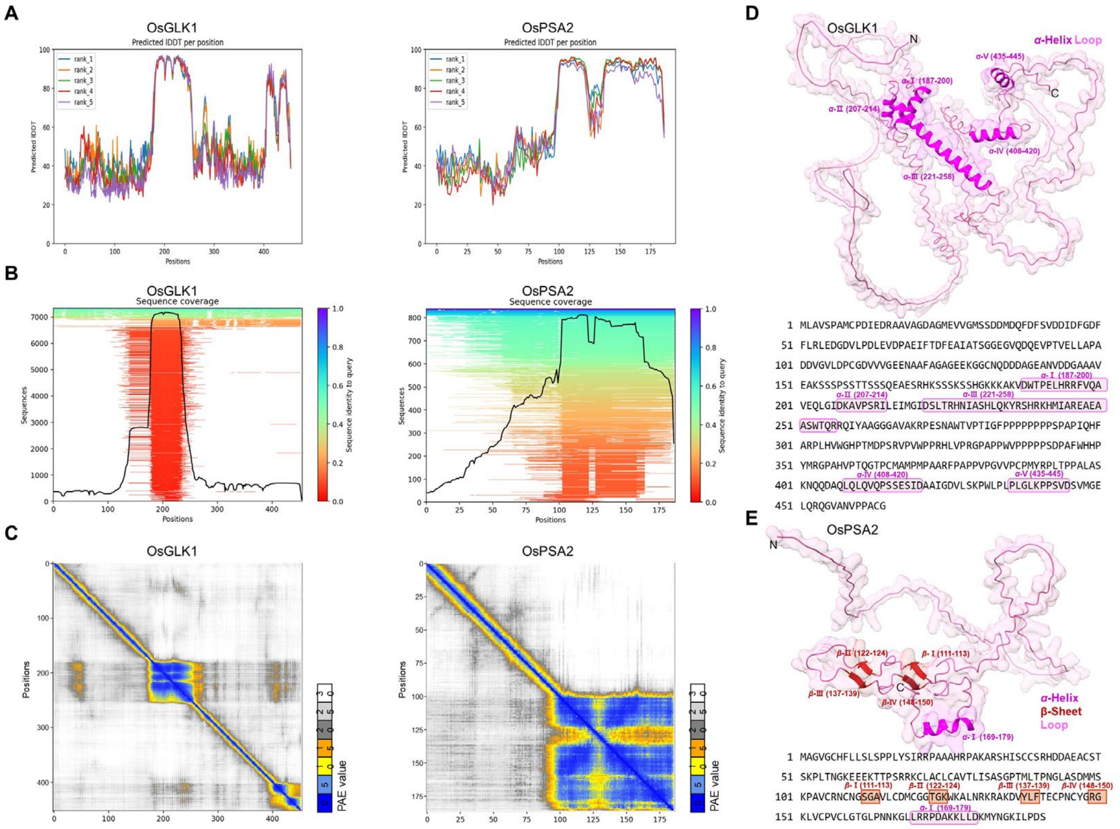

Supplementary Figure S9. 3D structure modeling of OsGLK1 and OsPSA2 (Supports Figure 5). **A** to **C)** The structural reliability and expected position error among OsGLK1 and OsPSA2 proteins were analyzed by lDDT **(A)**, MMseqs2 **(B)** and PAE plot **(C)**. The lDDT includes five top ranking structures and ranking #1 construct is used for further analysis. **D)** The 3D modeling of OsGLK1 was shown with the Myb domain (187-258) and tweezer-like C-term end (408-455), which includes three and two α-helix structures, respectively. **E)** The 3D modeling of OsPSA2 was shown with zinc finger domain (111-150), which includes four β-sheets.

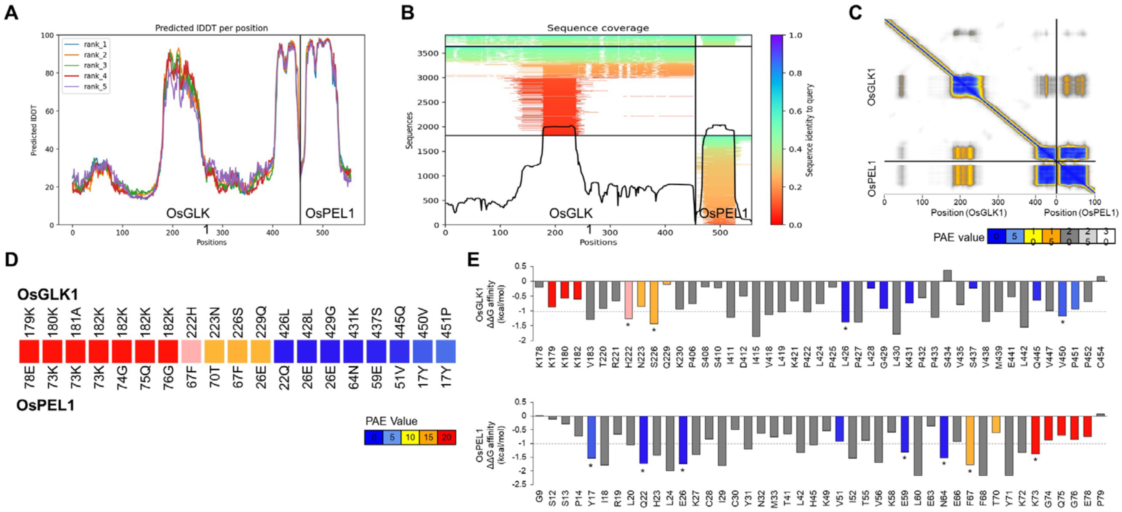

Supplementary Figure S10. Detailed information of 3D modeling from OsPEL1 and OsGLK1 complex shown in Fig. 5A. **A** to **C)** The structural reliability and expected position error among OsPEL1 and OsGLK1 proteins were tested by IDDT **(A)**, MMseqs2 **(B)** and PAE plot **(C)**. The IDDT includes five top ranking structures and ranking #1 construct is used for further analysis. **D** and **E)** Predicted amino acids for direct binding were shown by contact value from PAE plot **(D)** and alanine scanning **(E)**. The intersected amino acids were marked with color and asterisk in (E) and Fig. 5A.

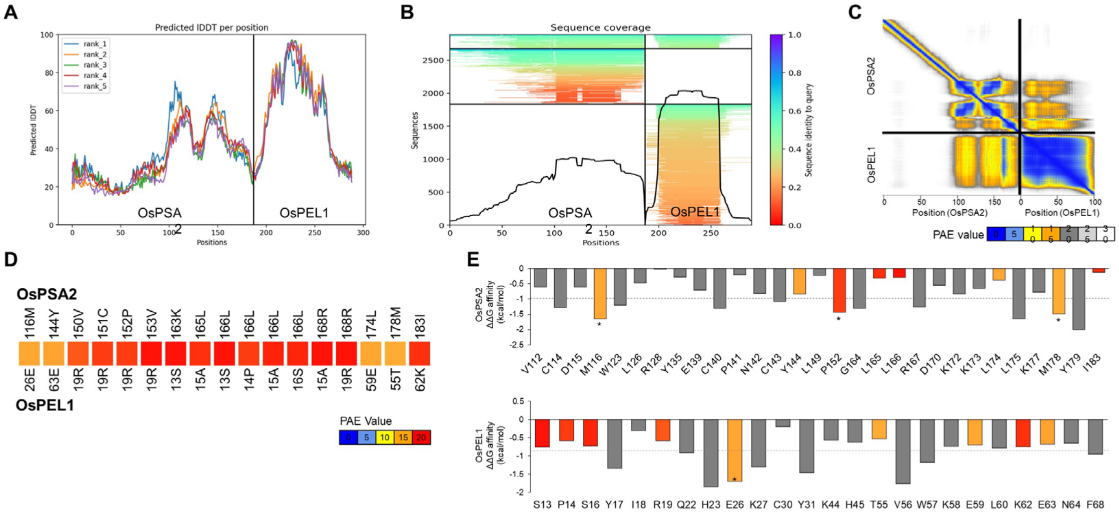

Supplementary Figure S11. Detailed information of 3D modeling from OsPEL1 and OsPSA2 complex shown in Fig. 5B. **A** to **C)** The structural reliability and expected position error among OsPEL1 and OsPSA2 proteins were tested by lDDT **(A)**, MMseqs2 **(B)** and PAE plot **(C)**. The lDDT includes five top ranking structures and ranking #1 construct is used for further analysis. **D** and **E)** Predicted amino acids for direct binding were shown by contact value from PAE plot **(D)** and alanine scanning **(E)**. The intersected amino acids were marked with color and asterisk in (E) and Fig. 5B.

Supplementary Data Set 1. Total gene lists of the PEL family in 64 photosynthetic plant species (Supports Figure 1).

Supplementary Data Set 2. FASTA (.txt) file of the alignment in Figure 1.

Supplementary Data Set 3. Newick (.nwk) file for phylogeny in Figure 1.

Supplementary Data Set 4. Quality control (QC) results of RNA-seq analysis (Supports Figure 8).

Supplementary Data Set 5. Significantly altered genes in the *Ospel1,2,3* compared to NT (Supports Figure 8).

Supplementary Data Set 6. Significantly altered genes in the *OsPEL1*-OE compared to NT (Supports Figure 8).

Supplementary Data Set 7. Detail fold change values of *PhANGs* and *PhAPGs* in Figure 8.

Supplementary Data Set 8. List of primers used in this study (Supports Figure 2,4,6,7 and 8).
